# Prediction of Postoperative Vomiting Within 24 Hours Using Machine Learning With Large Language Model–Enhanced Interpretability: Development and Validation Study

**DOI:** 10.2196/84260

**Published:** 2026-07-31

**Authors:** Huan-Jun Wang, Wei-Po Lee, Tz-Ping Gau, Kuang-I Cheng, Cheng-Ru Wei

**Affiliations:** 1 Department of Information Management, National Sun Yat-sen University, No. 70, Lianhai Rd., Gushan District, Kaohsiung 80424, Kaohsiung, Taiwan, 886 983337217; 2 Department of Anesthesiology, Kaohsiung Medical University Hospital, Kaohsiung, Taiwan

**Keywords:** postoperative vomiting, machine learning, clinical prediction model, perioperative risk prediction, electronic health records, interpretability, SHAP, large language models, clinical decision support, temporal validation, Shapley Additive Explanations

## Abstract

**Background:**

Postoperative nausea and vomiting are common complications after anesthesia. However, vomiting represents a clinically distinct and objectively measurable endpoint.

**Objective:**

This study aimed to develop and internally validate predictive models for postoperative vomiting within 24 hours using structured perioperative data and unstructured clinical text, while introducing a structured framework that separates feature construction from interpretability using large language models (LLMs).

**Methods:**

We analyzed 33,460 anesthesia records from a single center (2019‐2022). Two temporally defined prediction tasks were constructed to reflect real-world clinical decision-making and prevent information leakage: a preoperative model using variables available before anesthesia induction, and a perioperative model using variables available up to the end of surgery. Structured data were modeled using machine learning algorithms (logistic regression, Extreme Gradient Boosting, Light Gradient Boosting Machine [LightGBM]). Unstructured clinical text was incorporated through a deterministic, concept-driven preprocessing pipeline, where LLMs were used solely for normalization (temperature=0) without feature generation, followed by rule-based concept mapping and feature encoding. Post hoc interpretability was further supported using an LLM-based Question Answering Chain module. Model performance was evaluated using receiver operating characteristic-area under the curve (AUC), precision-recall AUC, calibration metrics, and threshold-based operating characteristics. Classification thresholds were selected using the Youden J statistic, and all metrics were reported with 95% CIs derived from bootstrap resampling. Decision curve analysis was performed to assess clinical utility.

**Results:**

A total of 33,460 surgical procedures were included, of which 3607 (10.8%) experienced postoperative vomiting within 24 hours. In the preoperative task, LightGBM achieved an AUC of 0.729 (95% CI 0.706‐0.749), compared with 0.610 (95% CI 0.588‐0.632) for the Apfel score. In the end-of-surgery task, LightGBM achieved an AUC of 0.735 (95% CI 0.714‐0.757). At the Youden-optimal threshold, the negative predictive value exceeded 0.95 across all models. Decision curve analysis demonstrated positive net benefit across clinically relevant threshold probabilities. Incorporating text-derived features provided modest improvements, while LLM-based explanation modules generated structured, natural-language explanations intended to enhance interpretability without substantially improving predictive performance.

**Conclusions:**

Machine learning models can effectively predict postoperative vomiting within 24 hours using perioperative data. The proposed framework demonstrates that LLMs can be integrated in a controlled and reproducible manner—restricted to deterministic normalization and post hoc reasoning—to generate natural-language explanations intended to enhance the interpretability of model predictions, without introducing information leakage or altering predictive modeling. As no formal clinician-based evaluation was conducted, this interpretability benefit cannot yet be objectively confirmed, and the generated explanations should be regarded as a useful interpretability aid to be validated in future clinician-centered studies. External, multicenter validation is required before broader clinical applicability can be assumed.

## Introduction

### Background

In clinical medicine, anesthesia is a fundamental component across a wide range of procedures. However, it is associated with several postoperative complications, among which postoperative nausea and vomiting (PONV) remains one of the most common and clinically significant. PONV can disrupt recovery, increase the risk of secondary complications, prolong hospitalization, and negatively impact patients’ perceived quality of life [1].

Accurate identification and stratification of PONV risk factors are essential for guiding preventive strategies. The overall incidence of PONV is estimated at 27.7%, underscoring its clinical importance [2]. Traditional risk assessment approaches primarily rely on static patient characteristics, such as sex, age, smoking status, and prior history of PONV or motion sickness [3,4]. However, these factors alone may be insufficient to capture the complexity of perioperative risk.

Although PONV includes both nausea and vomiting, these outcomes differ in mechanisms and clinical implications. Therefore, this study focuses specifically on postoperative vomiting within 24 hours (POV 24h) as a clearly defined and objectively measurable endpoint. Existing prediction tools, such as Apfel’s simplified risk score, report moderate accuracy (55%‐80%) [5], but are largely based on fixed patient characteristics and do not account for the temporal availability of perioperative information [6]. As a result, their applicability in real-time clinical decision-making remains limited.

Despite these advances, current prediction models face several key methodological limitations. First, most models do not explicitly distinguish between variables available at different clinical time points, potentially introducing information leakage by incorporating variables not available at the intended prediction time point, thereby limiting real-world clinical deployment. Second, perioperative factors—such as intraoperative physiological changes and anesthetic exposure—are often underrepresented despite their clinical relevance. Third, unstructured clinical text, including medical history and procedural notes, is rarely incorporated into predictive modeling due to integration challenges.

To address these gaps, we propose a temporally structured prediction framework that separates preoperative and perioperative modeling tasks. In addition, we explore the use of large language models (LLMs) in a structured framework that separates feature construction from interpretability to transform unstructured clinical text into structured representations and to support post hoc clinical reasoning, with the primary goal of generating structured explanations and supporting transparent post hoc review, rather than substantially improving predictive performance.

To evaluate this framework, we conducted a large-scale study using 33,460 anesthesia records, incorporating both structured (eg, physiological parameters, surgical, and anesthetic information) and unstructured data (eg, medical history and procedural notes). The objective of this study was to develop and internally validate predictive models for POV 24h. Two models were constructed based on the availability of clinical data: a preoperative model using variables available before anesthesia induction, and a perioperative model using variables available up to the end of surgery. Furthermore, we investigated whether LLM-based processing of unstructured clinical text could be performed while maintaining methodological rigor and supporting clinically grounded reasoning.

### Related Works

To overcome the limitations of conventional models, recent studies have increasingly turned to machine learning (ML) and deep learning (DL) techniques for PONV prediction [7]. These methods are well-suited for analyzing high-dimensional clinical datasets and capturing complex, nonlinear relationships that often elude traditional statistical models [8,9]. Although most prior studies focus on the composite outcome of PONV, vomiting can be assessed as a distinct and clinically measurable postoperative outcome within a defined time frame [10]. Therefore, this study specifically focuses on POV 24h.

Among these approaches, gradient boosting models like LightGBM (Light Gradient Boosting Machine) have been widely used to identify key PONV predictors and assist anesthesiologists in postoperative risk evaluation [11]. Common predictors highlighted across ML studies include sex, smoking status, type of surgical procedure, and use of opioids [12]. DL further advances prediction by modeling intricate interactions among features, with comparative analyses showing that algorithms such as logistic regression, support vector classifiers (SVCs), and AdaBoost frequently outperform traditional scoring systems [13]. Notably, TabNet, a DL architecture tailored for tabular data, leverages instance-level feature selection and attention mechanisms to improve efficiency and model transparency [14].

Despite these advances, the opaque nature of many ML and DL models presents a barrier to clinical adoption. To address this, explainable AI (XAI) techniques such as Shapley Additive Explanations (SHAP) have been introduced to illuminate the decision-making process of complex models. SHAP enables clinicians to assess the influence of individual features on prediction outcomes [15-17]. For instance, Jiang et al [18] demonstrated that incorporating traditional Chinese medicine (TCM) metrics alongside glycosylated hemoglobin levels substantially enhanced the interpretability of ML models in diabetic neuropathy. Similarly, in PONV research, SHAP has revealed previously unrecognized high-dimensional risk factors linked to delayed but clinically significant postoperative nausea and vomiting (CIPONV) [19].

Preoperative assessment and optimization have long been recognized as critical components in surgical care [20,21]. More recently, LLMs such as ChatGPT have been increasingly explored as tools to enhance the interpretability and usage of unstructured clinical text. LLMs have shown promise in preoperative evaluations and have even demonstrated alignment with expert consensus on pharmacologic strategies for PONV management [22-24]. However, current LLM implementations tend to function independently of structured patient data and often overlook patient-specific factors such as age, sex, and clinical history [22,24-26].

Past PONV research has laid a strong foundation for risk factor identification, offering essential insights for future feature engineering and data preprocessing efforts [27,28]. Nonetheless, in today’s fast-paced clinical environments, real-time decision-making is increasingly entrusted to frontline providers. Moreover, ML and DL systems should serve as enhancements to, and not replacements for, human clinical decision-making, especially in real-time environments where clinicians must rapidly interpret patient data [29].

Recent studies emphasize that beyond predictive performance, model interpretability and calibration are critical for clinical deployment. For instance, explainable tree-based models such as ExtraTrees have shown that even models with moderate discrimination require careful evaluation of calibration and feature contributions before clinical adoption, highlighting the importance of aligning model outputs with real-world clinical distributions and decision thresholds [30]. In addition, systematic reviews of clinical AI applications have identified persistent challenges in interpretability, bias, and insufficient validation, particularly in surgical and perioperative decision-making [31]. These concerns further underscore the need for greater transparency and trust in AI systems, as emphasized in broader discussions on AI deployment [32,33].

Taken together, future research should not only focus on improving predictive accuracy but also prioritize interpretable modeling, robust validation (including calibration), and transparency-aware model design, thereby fostering greater clinician confidence and facilitating real-world implementation.

## Methods

### Overview

**Figure 1.**
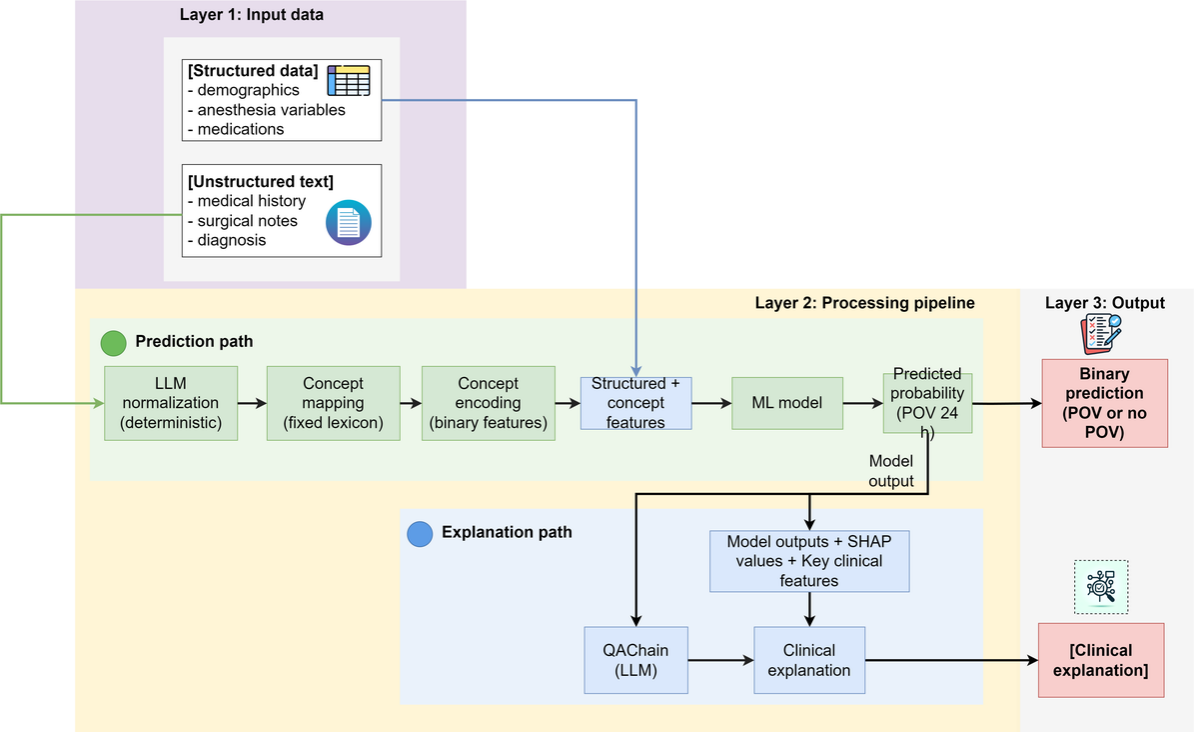
Proposed framework for predicting postoperative vomiting within 24 hours (POV 24h). The framework integrates modules for data preprocessing, model construction, and interpretability analysis. Traditional machine learning (ML) and deep learning (DL) methods serve as predictive baselines, while large language model (LLM)-based techniques support the structured representation of unstructured clinical data and generate post hoc explanations of model predictions. DL: deep learning; LLM: large-language model; ML: machine learning; POV: postoperative vomiting; QA: question answer.

We propose a clinically grounded framework for predicting POV 24h using ML, deep learning (DL), and LLMs. The framework comprises three core modules: data preprocessing, model development, and interpretability analysis (Figure 1). Traditional ML and DL models serve as predictive baselines, while LLM-based techniques support the structured transformation of unstructured clinical data and support post hoc interpretation within the modeling framework.

The primary endpoint was POV 24h, excluding nausea-only events. For each surgical case, multiple postoperative interviews were aggregated at the surgery level. A case was labeled positive (1) if any documented vomiting event occurred within 24 hours, and negative (0) if all available interviews indicated no vomiting. Surgeries without a valid 24-hour follow-up were excluded from the main analysis.

To reflect real-world clinical decision-making, we defined two prediction tasks based on the timing of feature availability: (1) a preoperative model using variables available before anesthesia induction, and (2) a perioperative model using variables available up to the end of surgery. Only variables available prior to each prediction time point were included in model training. Postoperative variables were excluded from all prediction models. To minimize the risk of temporal leakage, all variables were classified according to temporal availability prior to model construction (Multimedia Appendix 1). Variables unavailable at the intended prediction time point were excluded from the corresponding prediction task.

### Ethical Considerations

This retrospective study was approved by the Institutional Review Board of Kaohsiung Medical University Hospital (approval number: KMUHIRB-E(I)-20200040). The requirement for informed consent was waived due to the deidentified and retrospective nature of the data. The study was conducted in accordance with the principles of the Declaration of Helsinki.

The dataset was obtained from the hospital's electronic medical records system. Data collection and processing were conducted following institutional review board (IRB) approval (IRB No: KMUHIRB-E(I)-20200040).

### Structured Data Preprocessing

The dataset used in this study was obtained from a collaborating medical center and comprises both structured and unstructured data. Structured data includes physiological measurements, anesthetic information, and surgical procedure types. Unstructured data consists of narrative text, such as patient medical history and surgical records.

Structured data preprocessing was performed to ensure data quality and consistency prior to model development. An important characteristic of this dataset is that the hospital information system encodes the absence of an event or measurement as a value of 0 rather than as a conventional missing value (not a number [NaN]). For example, an intraoperative drug dosage of 0 indicates that the drug was not administered, and an airway-equipment size of 0 indicates that the corresponding procedure was not performed. Consequently, the structured feature set contained no conventional missing (NaN) values: across all 78 perioperative model features and the full study cohort (n=33,460), the NaN rate was 0%. A median- and mode-imputation step (for numeric and categorical variables, respectively) was nonetheless retained within the unified modeling pipeline as a safeguard for prospective deployment, where sporadic true missing values may occur; in the present study dataset this step was not triggered because no NaN values were present. All preprocessing procedures were refitted independently within each training fold before being applied to the corresponding validation data, ensuring that any imputation parameters would be derived exclusively from training data and preventing information leakage.

It is therefore important to distinguish these structurally coded zeros from genuine missing data. To characterize their distribution, we summarized the per-variable zero rate across the cohort. Most numeric features were near-completely recorded, whereas a small number of procedure-specific measurements (eg, airway-equipment sizes and selected intraoperative drug dosages) showed high zero rates that reflect non-performance of the corresponding procedure rather than missing information. A detailed per-variable zero-rate summary is provided as a supplementary file (Multimedia Appendix 2). Alternative imputation strategies, including K-Nearest Neighbors (KNN) imputation, were explored during preliminary assessment but did not materially affect downstream model performance and were therefore not adopted in the primary pipeline.

Extreme values were handled using percentile-based capping to reduce the influence of outliers while preserving clinically meaningful variation. Antiemetic administration was retained as a perioperative variable rather than excluded. As this variable may reflect clinicians’ responses to perceived risk and thus introduce potential confounding, prediction tasks were explicitly defined based on temporal availability, with variables included only when available at the time of prediction. The potential confounding effect of this variable was considered during the interpretation of model performance.

The study protocol was reviewed and approved by the Institutional Review Board (IRB) of Kaohsiung Medical University Hospital (approval number: KMUHIRB-E(I)-20200040). All data used for analysis were fully deidentified in accordance with institutional data protection and ethical standards. Direct identifiers (eg, patient names, national identification numbers, and dates of birth) were completely removed. For indirect identifiers (eg, admission time, hospital stay, and diagnosis codes), data masking or generalization techniques were applied to minimize the risk of reidentification.

### Unstructured Text Data Preprocessing

LLM-based methods for unstructured clinical text processing were implemented using a fixed model version with deterministic settings (temperature=0) to ensure reproducibility. All inputs to the LLM were strictly limited to deidentified text. Direct identifiers (eg, patient names, identification numbers, and exact dates) were completely removed prior to processing. When a commercial API was used, only deidentified text was transmitted through secure, encrypted channels in accordance with institutional data governance policies. No direct identifiers or personally identifiable information were transferred to external services. Alternatively, LLM processing could be conducted within a controlled or self-hosted environment. This design supports reproducible and secure use of LLM technologies within the clinical data pipeline while maintaining compliance with data protection regulations.

Handling unstructured clinical text requires approaches beyond conventional categorical encoding. We implemented a concept-based framework in which narrative text was normalized and mapped to predefined clinical concepts using LLM-assisted semantic standardization. Ordinal encoding was applied separately to ordered categorical variables, such as American Society of Anesthesiologists (ASA) physical status, by assigning ranked numerical values, enabling machine learning models to capture ordinal relationships effectively. However, more complex narrative text requires semantic interpretation that cannot be fully represented through predefined ordinal or categorical structures alone.

**Figure 2.**
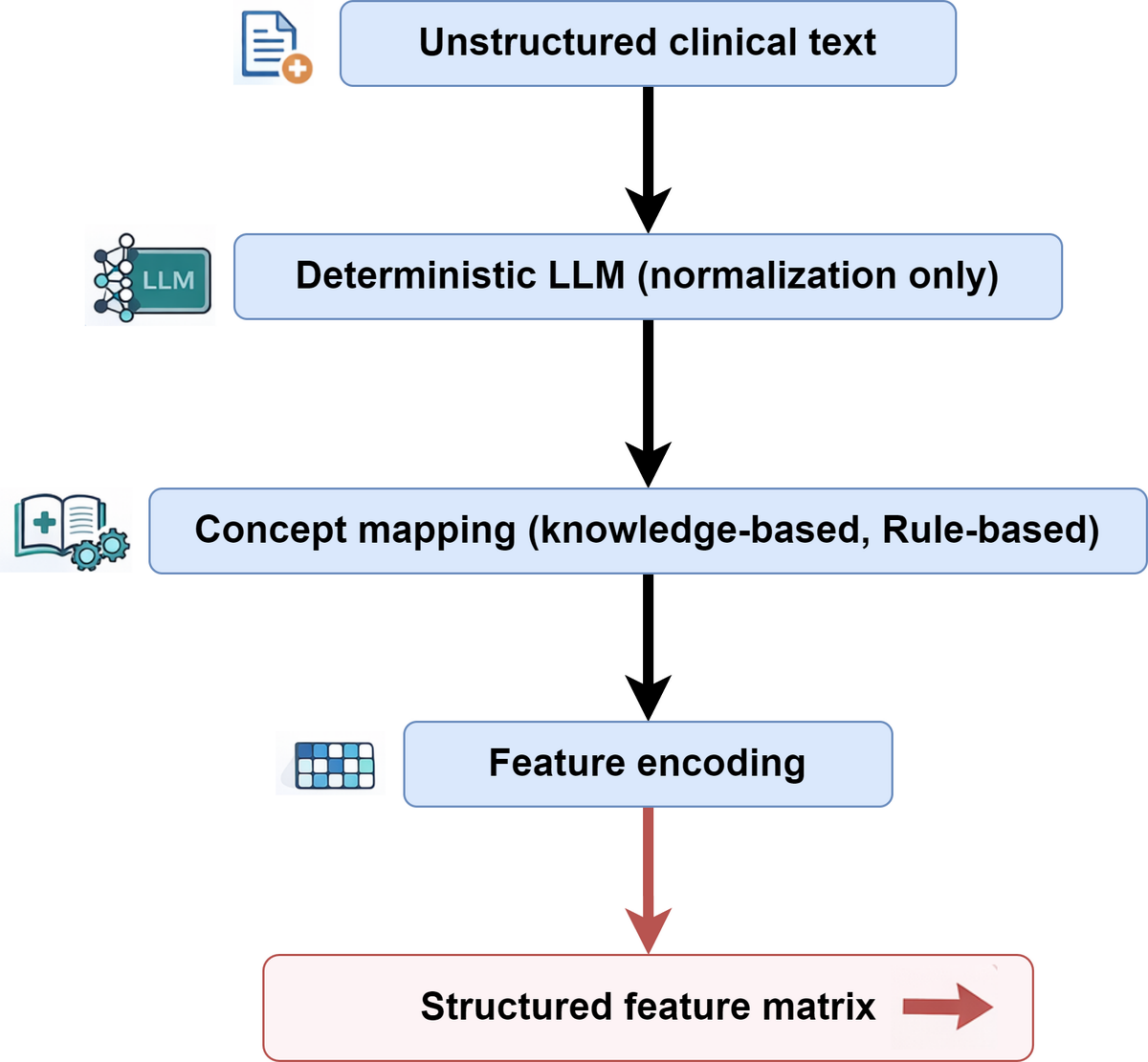
Clinical data preprocessing framework for unstructured text.

To extract clinically meaningful signals from medical history, surgical notes, and diagnostic descriptions, we implemented a keyword-based feature construction approach guided by predefined clinical concepts derived from prior literature and domain knowledge. The LLM was used solely to standardize semantically related expressions (eg, synonyms and linguistic variations) into consistent representations aligned with predefined clinical concepts. The identified concepts were subsequently mapped to structured features using a predefined scoring scheme, ensuring consistent and reproducible feature construction. The overall preprocessing pipeline for unstructured clinical text is illustrated in Figure 2.

Beyond keyword-based feature construction, we further incorporated Question Answering (QA) Chain, an LLM-based framework built on LangChain, as a post hoc explanation module to support clinical interpretation of model outputs. QAChain operates after model prediction by leveraging structured inputs, including model outputs and derived clinical features, to generate context-aware explanations. With a deterministic setting (temperature=0), the model produces consistent, structured explanations aligned with clinical reasoning and grounded in patient context.

**Figure 3.**
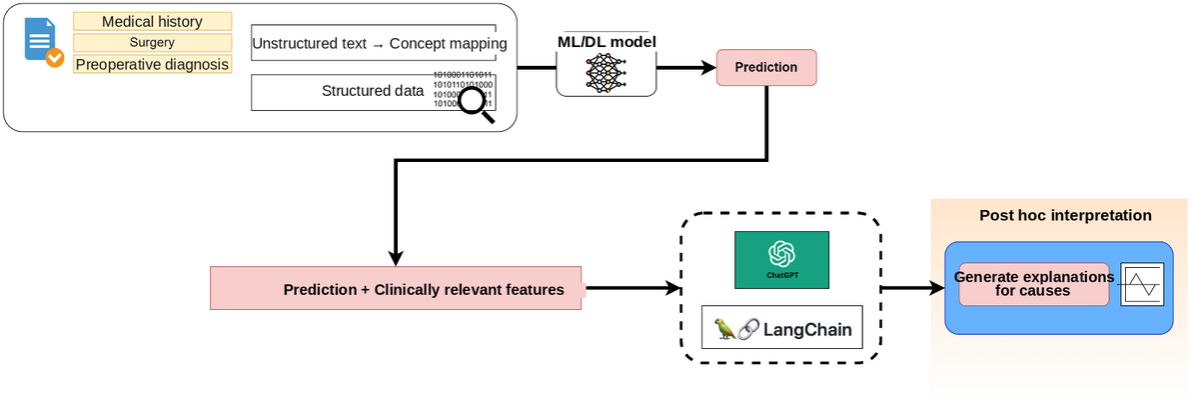
QAChain-based post hoc explanation framework. The QAChain module generates structured, natural-language explanations based on model predictions and input features. This module is used solely for interpretability purposes and does not participate in model training, feature construction, or prediction. DL: deep learning; ML: machine learning. QAChain: Question Answering Chain.

For example, it may generate: “The patient received high-dose opioids and underwent prolonged general anesthesia, both of which contribute significantly to PONV risk.” Such outputs provide interpretable narratives that connect model predictions with clinically relevant factors. The QAChain-based explanation process is illustrated in Figure 3. The full prompt template, system instructions, and configuration details are provided in Multimedia Appendix 3 to ensure reproducibility and transparency.

### Model Development

We developed predictive models for POV 24h using a temporally structured dual-task framework aligned with clinical decision-making. Two independent prediction tasks were constructed to prevent information leakage: (1) a preoperative model using variables available before anesthesia induction, and (2) a perioperative model using variables available up to the end of surgery. Only features available prior to each prediction time point were included in the corresponding model.

For structured data, three ML algorithms were implemented: logistic regression, LightGBM, and XGBoost (Extreme Gradient Boosting). Logistic regression served as an interpretable baseline model, while gradient boosting models (LightGBM and XGBoost) were used as primary predictive approaches due to their strong performance on tabular clinical data and their ability to capture nonlinear relationships and feature interactions.

Unstructured clinical text features derived from the preprocessing pipeline were incorporated into the model as structured inputs using a predefined keyword-based scoring system. This approach enabled integration of clinically relevant information from narrative data while maintaining methodological rigor and preventing information leakage.

Model training and evaluation were conducted under a temporal split design to simulate real-world deployment, in which models trained on earlier data were evaluated on unseen future cases. Model performance was assessed using discrimination (receiver operating characteristic–area under the curve and precision-recall-area under the curve), calibration (Brier score and calibration plots), and clinically relevant operating metrics. Classification thresholds were selected using Youden’s J statistic, and decision curve analysis was performed to evaluate clinical utility. All performance metrics were reported with 95% CIs derived from bootstrap resampling.

### Interpretability Analysis

To enhance transparency and support clinical interpretability, we incorporated both model-based and LLM-assisted explanation approaches. First, model-level interpretability was assessed using SHAP. SHAP values were used to quantify the contribution of individual features to model predictions and to derive global feature importance rankings based on the mean absolute SHAP values across samples. These analyses enabled the identification of key predictors associated with POV 24h.

Second, the keyword-based scoring system provided interpretable representations of unstructured clinical text, enabling examination of text-derived risk factors within the model.

Finally, we implemented QAChain, an LLM-based post hoc explanation module, to generate context-aware natural-language interpretations of model outputs. QAChain was configured with deterministic settings (temperature=0) to ensure reproducibility. Importantly, this module was not used for predictive modeling, but solely to provide human-readable explanations aligned with clinical reasoning.

Together, these approaches provide complementary perspectives on model behavior by combining quantitative feature attribution with qualitative explanation, thereby supporting clinical understanding and potential real-world application.

## Results

### Dataset

The study population consisted of patients who underwent anesthesia for surgical procedures at Kaohsiung Medical University Hospital between July 2019 and September 2022. The initial dataset included 79,919 anesthesia records comprising 127 variables and 95,304 postoperative interview records comprising 81 variables. These datasets were merged at the surgery level using a unique surgery identifier, resulting in a combined dataset with 208 variables.

**Figure 4.**
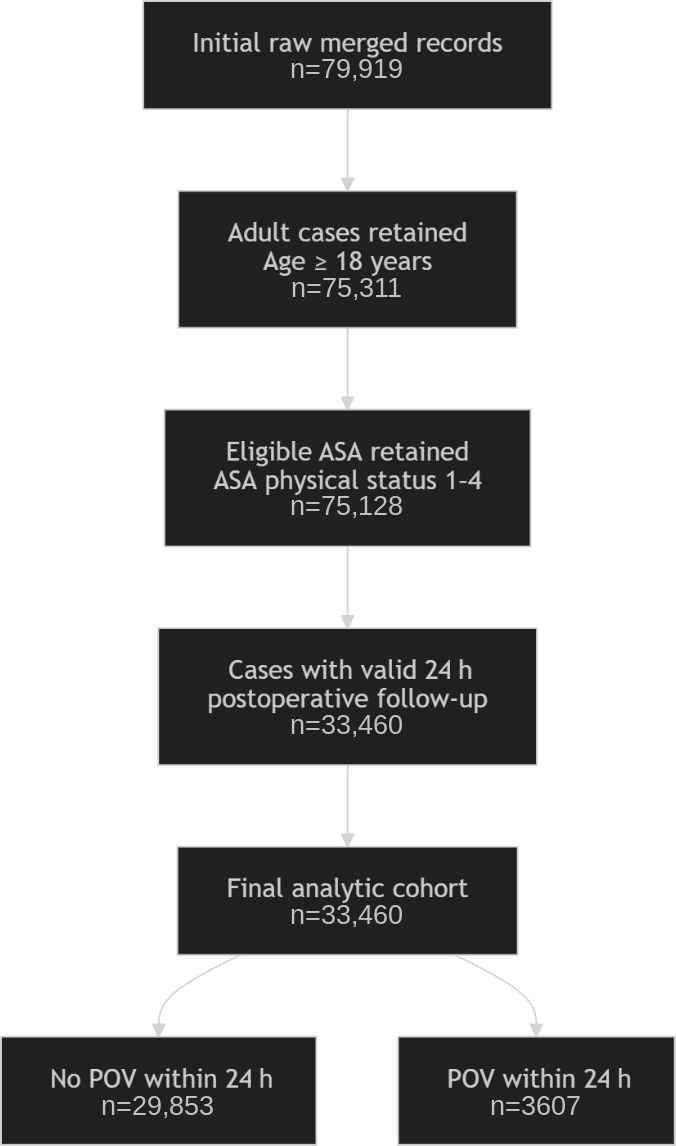
Cohort selection flow diagram.ASA: American Society of Anesthesiologists; POV: postoperative vomiting;

Cohort construction was performed based on predefined inclusion and exclusion criteria to ensure data quality and clinical consistency. Patients younger than 18 years, duplicate records, and cases with invalid or inconsistent timestamps were excluded. Patients with an ASA physical status of 5 or 6 were also excluded due to their extreme clinical risk profiles. In addition, cases without valid postoperative follow-up within 24 hours were excluded from the primary analysis. The cohort selection process is illustrated in Figure 4. (Multimedia Appendix 4)

Prophylactic antiemetic use was retained as a variable and further examined through sensitivity and subgroup analyses. After cohort construction, the final study population consisted of 33,460 surgical cases. Among these, 3607 cases (10.8%) experienced postoperative vomiting within 24 hours (POV 24h), while 29,853 cases (89.2%) did not.

The primary endpoint was defined as POV 24h. For each surgical case, postoperative interview records across multiple time windows were aggregated at the surgery level. A case was labeled as positive if vomiting occurred at any time within the 24-hour postoperative period.

Given the class imbalance in POV 24h outcomes, model evaluation focused on metrics that reflect both discrimination and clinical relevance, including receiver operating characteristic – area under the curve, precision-recall area under the curve, and operating characteristics such as sensitivity and positive predictive value.

### Model Performance

We evaluated three ML models (logistic regression, LightGBM, and XGBoost) for POV 24h. Logistic regression served as an interpretable baseline, while LightGBM and XGBoost were used to capture nonlinear relationships and feature interactions in tabular clinical data. The Apfel score was included as a clinical reference baseline.

All models were evaluated using a temporal split protocol, in which models were trained on earlier cases and tested on subsequent, unseen cases. Two prediction settings were defined according to clinical workflow: a preoperative model (using variables available before anesthesia induction) and a perioperative model (using variables available up to the end of surgery).

**Figure 5.**
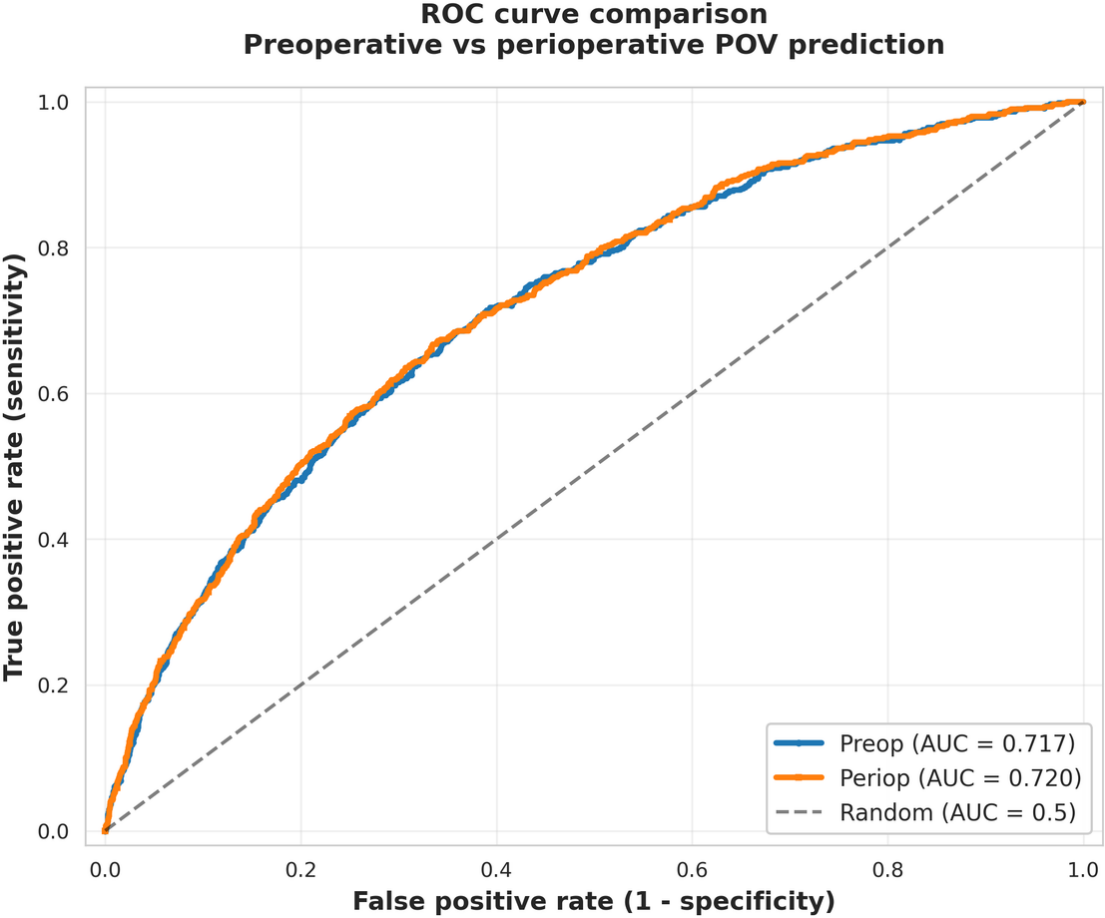
ROC curve comparison for preoperative and perioperative POV prediction models. AUC: area under the curve; POV: postoperative vomiting; ROC: receiver operating characteristic.

Table 1 summarizes model performance using only structured variables. Across both prediction settings, discrimination was consistent across model families. Gradient boosting models generally achieved slightly higher receiver operating characteristic-area under the curve and precision-recall-area under the curve than logistic regression, while logistic regression showed lower Brier scores, indicating better calibrated probability estimates. The receiver operating characteristic curves for the preoperative and perioperative prediction models are shown in Figure 5, demonstrating similar discrimination performance across the two settings, with only a marginal improvement when perioperative variables were included.

**Table 1.** Model performance using structured variables only Bootstrap-based 95% CIs (BCa, 1000 resamples) are reported for machine learning models.

Prediction task and model		ROC-AUC^a,b^ (95% CI)	PR-AUC^c^ (95% CI)	*F*_*1*_-score (95% CI)	Brier score (95% CI)
Preoperative					
Apfel score		0.606	0.122	0.040	0.097
Logistic regression		0.717 (0.695 to 0.739)	0.205 (0.176 to 0.230)	0.217 (0.180 to 0.251)	0.090 (0.086 to 0.094)
LightGBM^d^		0.722 (0.703 to 0.746)	0.201 (0.174 to 0.226)	0.278 (0.254 to 0.302)	0.173 (0.168 to 0.176)
XGBoost^e^		0.720 (0.700 to 0.742)	0.200 (0.176 to 0.223)	0.278 (0.256 to 0.300)	0.172 (0.168 to 0.175)
Perioperative					
Logistic regression		0.720 (0.700 to 0.741)	0.210 (0.177 to 0.234)	0.164 (0.128 to 0.198)	0.083 (0.079 to 0.088)
LightGBM		0.730 (0.707 to 0.751)	0.212 (0.184 to 0.238)	0.285 (0.262 to 0.307)	0.170 (0.166 to 0.174)
XGBoost		0.730 (0.708 to 0.752)	0.213 (0.184 to 0.238)	0.289 (0.262 to 0.313)	0.168 (0.164 to 0.172)

a ROC: receiver operating characteristic.

b AUC: area under the curve.

c PR: precision-recall.

d LightGBM: Light Gradient Boosting Machine.

e XGBoost: Extreme Gradient Boosting.

The transition from preoperative to perioperative prediction yielded only marginal improvements in discrimination metrics. Notably, changes in threshold-dependent metrics such as *F*_1_-score were inconsistent across models, reflecting differences in operating characteristics rather than overall predictive capability. This suggests that a substantial proportion of predictive information is already available prior to anesthesia induction, with intraoperative variables providing limited incremental value in overall model discrimination.

The relatively small performance differences between model types further indicate that, under the current feature set, model selection plays a secondary role compared to the availability and timing of clinical information. The Apfel score, a rule-based clinical model based on four binary predictors, showed lower discrimination and *F*_1_-scores than ML models.

**Figure 6.**
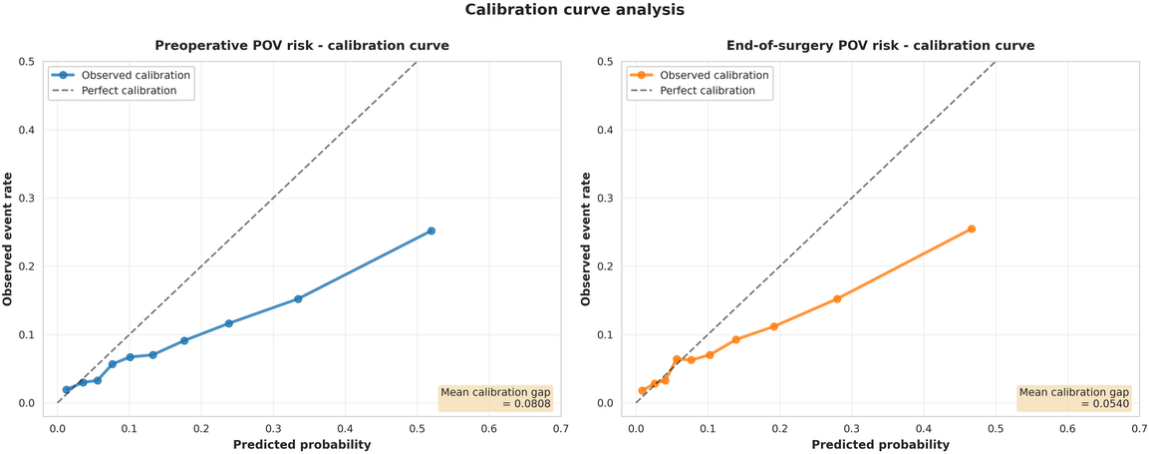
Calibration curves for preoperative and perioperative POV prediction models. POV: postoperative vomiting.

Calibration curves for both preoperative and perioperative models are presented in Figure 6. Overall, predicted probabilities showed reasonable agreement with observed event rates, although some deviation from perfect calibration was observed at higher predicted risk levels. This is consistent with the Brier score results reported in Table 1, which show that logistic regression demonstrated slightly better calibration performance. Clinical performance metrics at the Youden-optimal threshold are summarized in Table 2. The between-model differences in calibration carry practical implications for the intended clinical use. Because the framework is intended to support selective antiemetic prophylaxis—where action depends on whether a patient’s estimated risk exceeds an institution-specific threshold—well-calibrated probabilities are as important as discrimination. Logistic regression produced the lowest Brier scores and the closest agreement between predicted and observed risk, whereas the gradient boosting models, despite marginally higher discrimination, tended to overestimate risk at the highest predicted-probability levels. This suggests that, when absolute risk estimates rather than rank ordering alone are used to guide prophylaxis, either the better-calibrated logistic regression model or a post hoc recalibration of the gradient boosting models (eg, Platt scaling or isotonic regression) would be preferable.

**Table 2.** Clinical performance of machine learning models at the Youden-optimal threshold.

Model	Threshold	Sensitivity	Specificity	PPV^a^	NPV^b^	Youden J
Preoperative						
Logistic regression	0.17	0.600	0.733	0.179	0.950	0.333
LightGBM^c^	0.48	0.613	0.741	0.188	0.952	0.355
XGBoost^d^	0.44	0.671	0.678	0.168	0.955	0.348
Perioperative						
Logistic regression	0.13	0.635	0.712	0.177	0.952	0.347
LightGBM	0.50	0.593	0.762	0.195	0.951	0.355
XGBoost	0.44	0.664	0.683	0.169	0.954	0.347

a PPV: positive predictive value.

b NPV: negative predictive value.

c LightGBM: Light Gradient Boosting Machine.

d XGBoost: Extreme Gradient Boosting.

We further evaluated the contribution of unstructured clinical text by incorporating deterministic text-derived scores as structured model inputs. Table 3 presents model performance after including text-derived features. Across models, the inclusion of text-derived features resulted in modest changes in discrimination metrics, with slight improvements observed in several model and task settings. The magnitude of improvement was limited, indicating that structured perioperative variables account for the majority of the predictive signal.

**Table 3.** Model performance with text-derived features Bootstrap-based 95% CIs (bias-corrected and accelerated [BCa], 1000 resamples) are reported for machine learning models.

Prediction task and model		ROC-AUC^a^^b^^,^^b^ (95% CI)	PR-AUC^b^^,^^c^ (95% CI)	*F*_1_-score (95% CI)	Brier score (95% CI)
Preoperative					
Logistic regression		0.719 (0.699 to 0.742)	0.212 (0.183 to 0.238)	0.209 (0.173 to 0.245)	0.087 (0.082 to 0.092)
LightGBM^d^		0.722 (0.701 to 0.745)	0.210 (0.181 to 0.236)	0.284 (0.262 to 0.309)	0.172 (0.168 to 0.176)
XGBoost^e^		0.725 (0.701 to 0.745)	0.209 (0.184 to 0.235)	0.285 (0.264 to 0.309)	0.171 (0.168 to 0.175)
Perioperative					
Logistic regression		0.724 (0.704 to 0.746)	0.215 (0.188 to 0.244)	0.171 (0.137 to 0.206)	0.083 (0.078 to 0.087)
LightGBM		0.731 (0.709 to 0.750)	0.220 (0.190 to 0.244)	0.287 (0.261 to 0.308)	0.169 (0.166 to 0.173)
XGBoost		0.732 (0.710 to 0.752)	0.222 (0.192 to 0.248)	0.287 (0.262 to 0.312)	0.168 (0.164 to 0.172)

a ROC: receiver operating characteristic.

b AUC: area under the curve.

c PR: precision-recall.

d LightGBM: Light Gradient Boosting Machine.

e XGBoost: Extreme Gradient Boosting.

Text-derived features appear to provide complementary information, enriching the representation of clinical context without substantially altering overall model performance.

### Decision Curve Analysis

**Figure 7.**
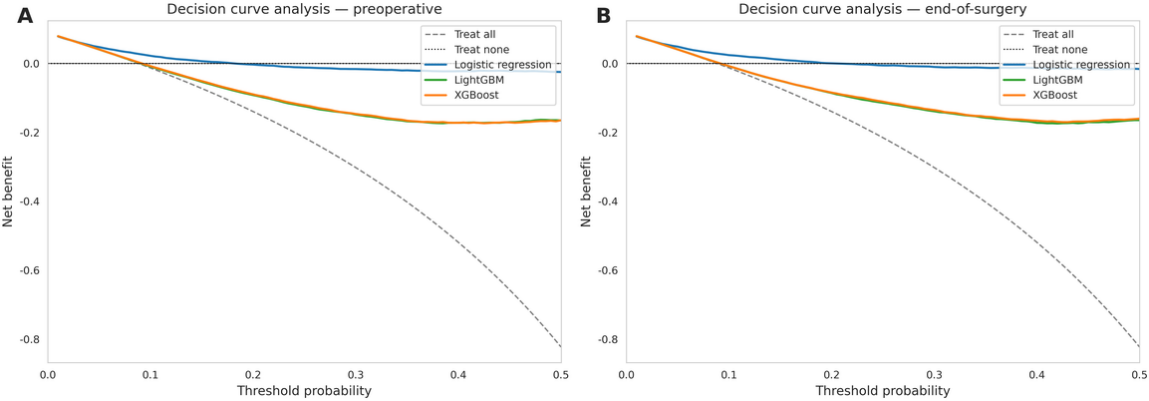
Decision curve analysis preoperative and perioperative postoperative vomiting (POV) prediction models. (A) Preoperative prediction models. (B) End-of-surgery prediction models. Net benefit is plotted against threshold probability for logistic regression, LightGBM, and XGBoost models, along with treat-all and treat-none reference strategies. LightGBM: Light Gradient Boosting Machine; XGBoost: Extreme Gradient Boosting.

Decision curve analysis was performed to evaluate the predictive models’ clinical utility across a range of threshold probabilities (Figure 7). For both preoperative and perioperative prediction tasks, the machine learning models demonstrated positive net benefit across clinically relevant threshold probabilities, supporting the potential value of model-guided selective prophylaxis strategies compared with treat-all or treat-none approaches.

### Interpretability Analysis

To enhance transparency and support clinical interpretation, we adopted a multi-level interpretability approach integrating model-based feature attribution, text-derived feature representation, and LLM-assisted explanation. SHAP was used to quantify feature contributions at the model level, the deterministic keyword-based scoring system provided traceability for unstructured clinical text, and QAChain generated context-aware, human-readable explanations of model predictions. Together, these approaches offer complementary insights into model behavior and support clinically meaningful interpretation.

### SHAP Analysis

**Figure 8.**
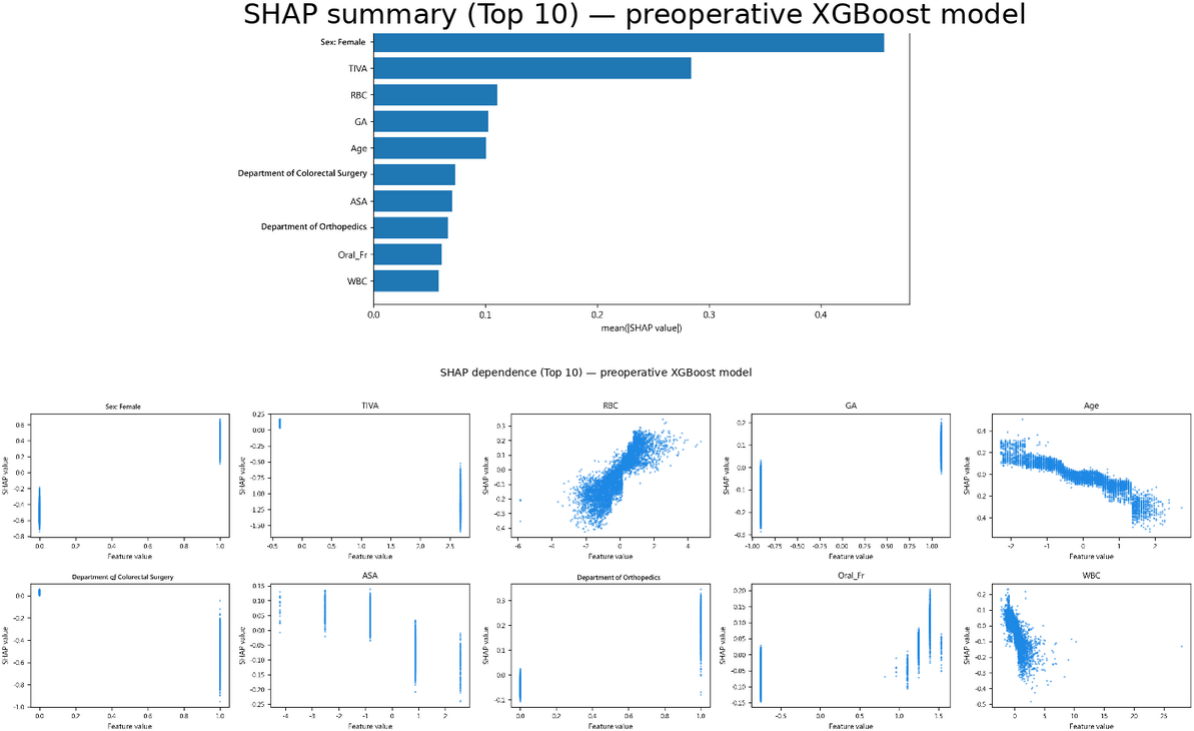
Combined SHAP summary and dependence plots for the XGBoost preoperative model. The summary bar plot displays global feature importance based on mean absolute SHAP values, while the dependence plots illustrate the direction and magnitude of each feature’s effect on prediction outcomes. Female sex, red blood cells (RBC), and general anesthesia are associated with increased predicted postoperative nausea and vomiting (PONV) risk, whereas total intravenous anesthesia (TIVA) shows a strong protective effect. Age demonstrates an inverse relationship with risk. Other variables, such as American Society of Anesthesiologists (ASA) status and surgical specialties, exhibit more complex patterns. GA: general anesthesia: PONV: postoperative nausea and vomiting: RBC: red blood cells: SHAP: Shapley Additive Explanations: TIVA: total intravenous anesthesia: WBC: white blood cells; XGBoost: Extreme Gradient Boosting.

To interpret the model’s internal decision-making mechanism, SHAP analysis was performed on the XGBoost model, which achieved the best overall predictive performance. Figure 8 presents the combined SHAP summary and dependence plots for the preoperative model. The summary plot ranks features according to their global importance based on mean absolute SHAP values, while the dependence plots further illustrate the direction and magnitude of each feature’s effect on the predicted PONV risk.

The most influential features include female sex, total intravenous anesthesia (TIVA), red blood cells, general anesthesia, age, ASA status, and selected surgical specialties. Among these, several variables exhibit clear, clinically meaningful directional patterns. Female sex shows a strong positive association with predicted PONV risk, with consistently higher SHAP values observed in female patients. In contrast, TIVA exhibits a substantial protective effect, with markedly negative SHAP values indicating reduced predicted risk. Age demonstrates an inverse relationship, where younger patients are associated with higher predicted risk, as reflected by decreasing SHAP values with increasing age.

Continuous variables, such as red blood cells, show a positive trend, indicating that higher values are associated with a higher predicted risk. Similarly, general anesthesia is associated with higher prediction outputs compared to nongeneral anesthesia approaches. Other variables, including ASA status and surgical specialties, exhibit more complex or nonlinear patterns, suggesting potential interactions or confounding effects within the model. These features contribute to prediction but require cautious interpretation.

**Figure 9.**
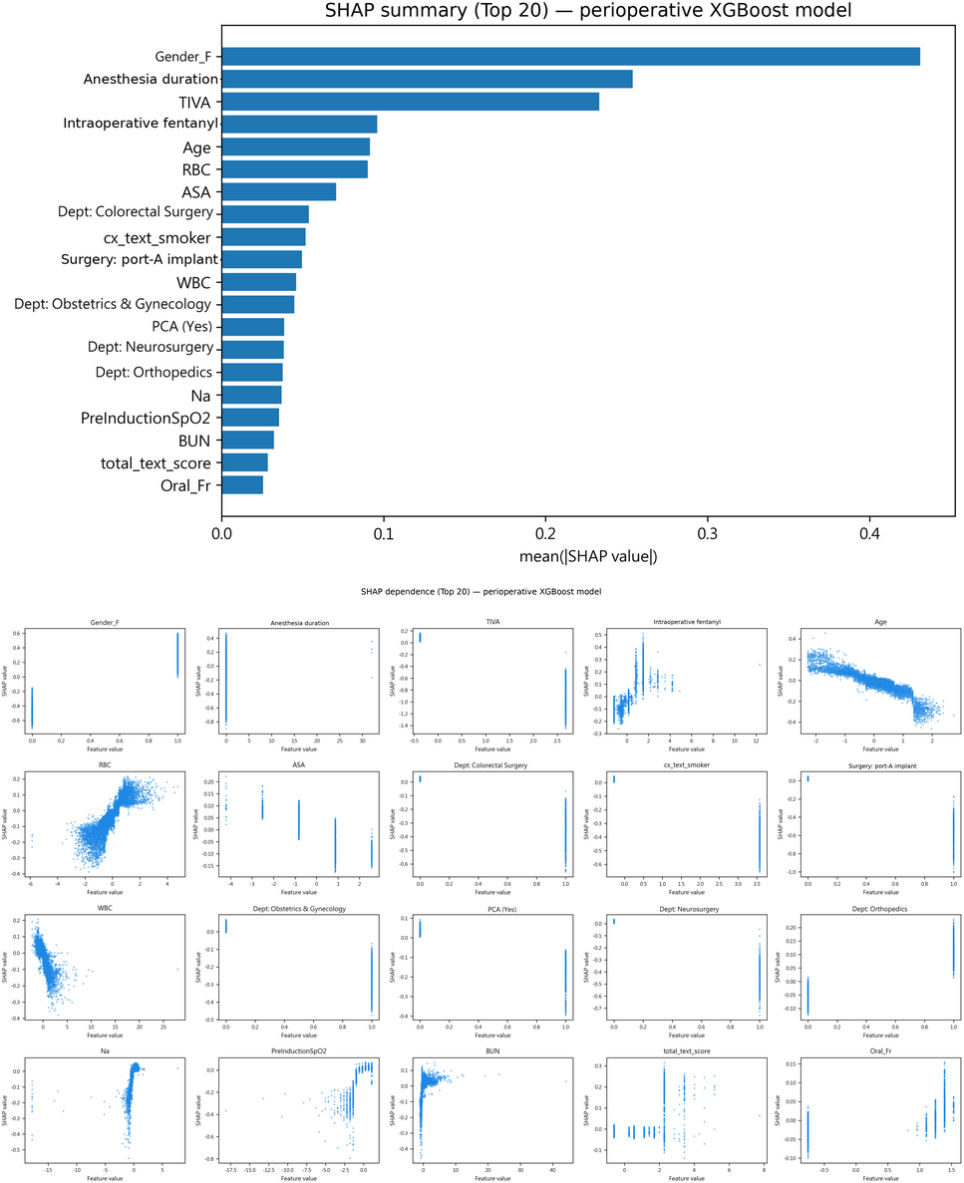
SHAP summary and dependence plots for the perioperative XGBoost model. The summary plot (top) presents the global importance of features ranked by mean absolute SHAP values, while the dependence plots (bottom) illustrate the relationship between feature values and their contributions to model predictions. Key predictors include sex, anesthesia duration, TIVA, opioid use (fentanyl), age, and laboratory variables. Text-derived features, including concept-based indicators (eg, cx_text_smoker) and procedure-specific features (eg, port-a-catheter implantation), also contribute to model predictions, although their influence is smaller compared to primary clinical variables. BUN: blood urea nitrogen; PCA: patient-controlled analgesia; RBC: red blood cells; SHAP: Shapley Additive Explanations; TIVA: total intravenous anesthesia; WBC: white blood cells; XGBoost: Extreme Gradient Boosting.

Overall, the SHAP analysis indicates that demographic characteristics, anesthetic strategies, and perioperative physiological variables jointly drive model predictions, with several features demonstrating consistent and interpretable directional effects aligned with clinical knowledge. Similar patterns were observed in the perioperative model (Figure 9), with additional contributions from intraoperative variables such as anesthesia duration and opioid use.

A structured summary of the top features, including their direction and clinical interpretation, is provided in Table 4.

**Table 4.** Summary of top SHAP features with direction and clinical interpretation.

Feature	Direction	Clinical interpretation
Preoperative model		
Female sex	↑	Strong established risk factor for PONV^a^
TIVA^b^	↓	Protective compared to general anesthesia
RBC^c^	↑	May reflect physiological status; associated with increased risk
GA^d^	↑	Known to increase PONV risk vs non-GA
Age	↓	Younger patients have higher risk
Colorectal surgery	↑	Procedure-related risk variation
ASA^e^	Nonlinear	Reflects overall health; nonlinear effect
Orthopedic surgery	Nonlinear	Procedure-specific variation
Oral_Fr	↑	Airway-related procedural factor
WBC^f^	↓	Weak or indirect association
Perioperative model		
Female sex	↑	Strong risk factor
Anesthesia duration	↑	Longer exposure increases risk
TIVA^b^	↓	Protective anesthetic strategy
Fentanyl (intraop)	↑	Opioid-related risk increase
Age	↓	Younger patients at higher risk
RBC^c^	↑	Physiological association
ASA	Nonlinear	Reflects baseline health
Smoking (text-derived)	↓	Smoking paradox (known effect)
Surgery type (colorectal)	↑	Procedure-related risk
Total_text_score	↑	Aggregated clinical context

a PONV: postoperative nausea and vomiting.

b TIVA: total intravenous anesthesia.

c RBC: red blood cell.

d GA: general anesthesia.

e ASA: American Society of Anesthesiologists.

f WBC: white blood cell.

### Text-Derived Feature Analysis

To connect the preprocessing pipeline with model interpretability, we examined how text-derived features constructed from LLM-assisted normalization and concept-based mapping contribute to model predictions.

The LLM was applied to selected unstructured clinical text fields, including medical history descriptions, surgical notes, and diagnostic narratives. These fields contain heterogeneous linguistic expressions, such as synonyms, abbreviations, and mixed-language representations, which cannot be reliably standardized using simple rule-based methods. Using a deterministic configuration (temperature=0), the LLM was employed to normalize semantically equivalent expressions into consistent representations aligned with predefined clinical concepts (eg, smoking status, prior PONV, and laparoscopic procedures). Importantly, the LLM was not used to generate new features or discover additional concepts, and no outcome-related information was used in this process. Only text fields available prior to the prediction time point were included, ensuring temporal consistency and preventing information leakage.

Clinically relevant concepts were predefined based on established literature and domain knowledge. Following normalization, clinical narratives were mapped to structured features using deterministic and rule-based procedures. Three types of text-derived features were constructed: (1) concept-based binary indicators (cx_text_*), representing the presence or absence of clinically meaningful conditions; (2) aggregated scoring features (eg, history_score, surgery_score, diagnosis_score, and total_text_score), derived from predefined weighting schemes; and (3) count-based features based on keyword frequency. While all final features are constructed deterministically, the LLM enhances the consistency and robustness of concept extraction from unstructured text.

Within the XGBoost model, SHAP analysis (Figure 9) shows that text-derived contributions are concentrated in a small number of concept-based features. In particular, cx_text_smoker and cx_text_ponv_history show substantially higher contributions than most other text-derived features, indicating that clinically meaningful concept indicators account for the majority of the text-derived signal in the model. In comparison, composite scoring variables (eg, history_score, surgery_score, diagnosis_score) show relatively limited contribution, while total_text_score provides a moderate but secondary contribution.

In addition to concept-based features, certain procedure-specific text-derived features (eg, port-a-catheter implantation) also appear among the top contributors, reflecting localized clinical patterns captured from surgical narratives that are not fully represented by predefined concept categories.

To further examine how the model uses different representations of clinical text, we grouped text-derived features into concept-based, aggregated-score, and count-based categories and visualized their SHAP contributions (Figure 9). This analysis shows that the model primarily relies on concept-based indicators, with aggregated- and count-based representations playing a secondary role.

Overall, these findings indicate that LLM-assisted semantic normalization enables consistent extraction of clinically meaningful concepts, which the model subsequently leverages as structured features. This design supports integrating unstructured clinical text into predictive modeling while preserving interpretability and reproducibility. Additional supporting analyses of text-derived features, including concept hit rates, postoperative vomiting rate comparisons, signal strength, the coverage funnel, and concept-level statistics, are provided in (Multimedia Appendix 5).

### QAChain Reasoning Analysis

To evaluate the interpretability and reliability of the proposed framework, we conducted a comprehensive analysis of QAChain, an LLM-based post hoc explanation module that generates context-aware, human-readable interpretations of model predictions.

At the case level, QAChain consistently produced clinically meaningful explanations aligned with patient-specific risk profiles. For high-risk cases, explanations frequently emphasized well-established risk factors such as opioid exposure (eg, intraoperative fentanyl), prolonged anesthesia duration, and general anesthesia. For instance, explanations often highlighted that high-dose opioid administration and extended anesthesia duration jointly contribute to increased PONV risk. In contrast, for low-risk cases, QAChain tended to emphasize protective or mitigating factors, such as the use of TIVA and shorter procedural duration. For intermediate or borderline cases, QAChain generated balanced explanations that incorporated both risk-enhancing and risk-reducing factors, reflecting nuanced and context-sensitive reasoning.

To quantitatively assess whether QAChain explanations align with model behavior, we evaluated their alignment with SHAP-based feature attributions, which serve as a proxy for model-derived feature importance. Specifically, for each case, the top-k features ranked by SHAP values were compared with the features explicitly referenced in the corresponding QAChain explanation. Alignment was defined as the proportion of SHAP top-k features that were mentioned in the generated explanation.

**Figure 10.**
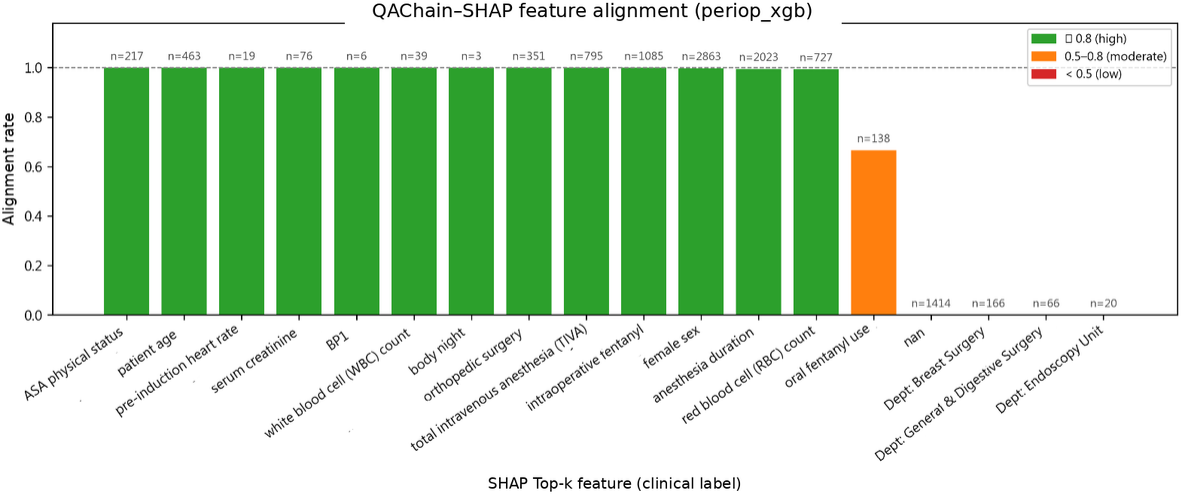
Feature-level alignment between QAChain explanations and SHAP attributions (perioperative XGBoost model). Each bar represents the proportion of cases in which a SHAP top-k feature is explicitly referenced in the corresponding QAChain-generated explanation. Numbers above bars indicate feature occurrence frequency. Most clinically relevant features align nearly perfectly, demonstrating that QAChain explanations are strongly grounded in model-derived feature importance. ASA: American Society of Anesthesiologists; SHAP: Shapley Additive Explanations; TIVA: total intravenous anesthesia; RBC: red blood cell; XGBoost: Extreme Gradient Boosting.

As illustrated in Figure 10, QAChain explanations show high consistency with model-derived feature importance at the surface level. Most clinically relevant features—such as intraoperative fentanyl, TIVA, anesthesia duration, patient age, and female sex—are frequently reflected in QAChain explanations, achieving near-perfect alignment rates (≈99%‐100%). For example, high-frequency features such as female sex (n=2863) and anesthesia duration (n=2023) exhibit alignment rates above 99%, indicating that QAChain reliably captures dominant predictive signals even in large-scale clinical data.

However, this alignment metric captures lexical feature mention rather than direct causal faithfulness. Therefore, the results should be interpreted as surface-form concordance between QAChain explanations and SHAP-derived feature importance, rather than definitive evidence of full mechanistic fidelity. Features with lower alignment, such as oral fentanyl use, likely reflect semantic abstraction, where QAChain generalizes specific variables into broader clinical concepts (eg, opioid exposure) rather than consistently preserving fine-grained distinctions. Additionally, variables such as missing values (“nan”) and categorical surgical department labels are rarely mentioned, suggesting that QAChain selectively prioritizes clinically interpretable and semantically meaningful features in its natural-language explanations.

Overall, these findings indicate that QAChain produces stable, clinically coherent explanations that are meaningfully related to model attributions, while allowing semantic abstraction and human-readable reasoning.

To ensure reproducibility, QAChain was configured with deterministic settings (temperature=0), resulting in highly consistent outputs across repeated runs with identical inputs. Furthermore, QAChain explanations were generated from structured, engineered features derived from the modeling pipeline, without direct access to raw unstructured text or outcome variables. This design reduces the risk of information leakage and helps ensure that explanations remain aligned with the model’s input space.

Collectively, these findings indicate that QAChain produces stable explanations that are internally consistent with model-derived signals and are intended to enhance the interpretability of model predictions. Because no formal clinician-based evaluation was performed, this interpretability benefit cannot be objectively confirmed at this stage; the explanations should therefore be regarded as a useful interpretability reference to be validated in future clinician-centered studies.

## Discussion

This study developed and internally validated a temporally structured clinical decision-support framework for POV 24h using ML and LLM-assisted interpretability methods. The results show that model performance was comparable across algorithms, with gradient boosting models demonstrating slightly higher discrimination and logistic regression showing better calibration. Most predictive information was already available before anesthesia induction, suggesting that clinically actionable risk stratification may be feasible during preoperative assessment and could support earlier prophylaxis planning and perioperative risk management. The limited incremental gain from perioperative variables suggests that a substantial proportion of clinically relevant risk information is already available before surgery. In addition, text-derived features contributed limited but consistent improvements, suggesting a complementary role. Finally, integrating SHAP-based attribution and LLM-based explanation enabled a multi-level interpretability framework that supports a clinically meaningful understanding of model predictions.

Building upon these findings, this study provides a deeper analysis of a temporally structured framework for POV 24h, integrating structured clinical variables, text-derived features, and multi-level interpretability methods.

The inclusion of text-derived features yielded consistent but limited performance gains, suggesting that unstructured clinical text provides complementary rather than dominant predictive signals. Importantly, the proposed LLM-assisted preprocessing approach enables consistent extraction of clinically meaningful concepts from heterogeneous narrative data while preserving interpretability and reproducibility.

From an interpretability perspective, combining SHAP-based feature attribution with QAChain-generated explanations provides a multi-level view of model behavior. SHAP offers quantitative insight into feature contributions, while QAChain translates these signals into human-readable clinical narratives. The alignment analysis suggests that QAChain explanations are meaningfully related to dominant model signals, though the evaluation is based on lexical features and does not establish full causal faithfulness. Instead, QAChain can be interpreted as a translation layer that bridges structured feature attribution and clinically interpretable reasoning. We note that this characterizes the explanations generated by the framework; the extent to which they enhance clinician interpretability or usability remains to be confirmed through future clinician-based evaluation.

These findings position the proposed framework as a practical approach for enhancing the interpretability of clinical machine learning systems through multi-level explanation, without relying on a single explanation modality. Because a formal clinician-based evaluation was not conducted, the magnitude of this interpretability benefit could not be objectively quantified in the present study and remains to be confirmed through future clinician-centered evaluation.

Importantly, the role of LLMs in this framework should be interpreted as a deliberate design choice rather than a strategy aimed at maximizing predictive performance. While the inclusion of text-derived features resulted in only modest improvements, this reflects the dominance of structured perioperative variables in this prediction task rather than a limitation of the approach.

Instead, LLMs were intentionally constrained to deterministic semantic normalization and post hoc explanation rather than being used as autonomous predictive generators, thereby minimizing the risk of introducing additional predictive signal or potential information leakage. Under this design, QAChain functions as a translation layer that converts model-derived feature attributions into clinically interpretable narratives, rather than acting as an independent predictive component.

This design aligns with emerging directions in clinical AI, where interpretability, transparency, and alignment with clinical reasoning are increasingly recognized as essential for real-world deployment. From this perspective, the limited performance gain associated with LLM integration should be interpreted as evidence that explanations intended to enhance interpretability can be generated without compromising model validity, with the magnitude of this interpretability benefit remaining to be confirmed through future clinician-based evaluation.

Several limitations should be noted. First, the alignment analysis between QAChain explanations and SHAP features is based on surface-form matching, which may not fully capture semantic equivalence or causal reasoning. Second, QAChain explanations are generated using a fixed prompt and deterministic settings, which may limit variability but do not guarantee true interpretive fidelity. Third, this study is based on retrospective data from a single medical center, which limits external generalizability. Multicenter validation of POV prediction models is inherently challenging due to the heterogeneity of institutional anesthesia practices, antiemetic protocols, and postoperative interview procedures across sites, which can substantially alter both feature distributions and outcome definitions. Furthermore, standardized multi-center data sharing in perioperative research remains limited by privacy regulations and the absence of unified electronic health record schemas. These structural barriers make single-center development studies a common and accepted starting point in this domain. External validation in independent, multi-center cohorts nevertheless remains an essential prerequisite before broader clinical applicability of the proposed models can be assumed, and we caution against generalizing the present single-center findings to other settings without such validation. In addition, the clinical usefulness of QAChain-generated explanations was not formally evaluated by clinicians in this study. Conducting a prospective clinician assessment requires a dedicated deployment environment and institutional review board approval beyond the scope of the current retrospective study. The alignment analysis between QAChain explanations and SHAP attributions provides an objective proxy for consistency, but this does not substitute for human-centered evaluation. Clinician-based usability studies are therefore explicitly identified as a priority for future research.

### Conclusion

This study presents a temporally structured framework for POV 24h that integrates structured clinical variables, text-derived features, and multilevel interpretability methods. By explicitly separating preoperative and perioperative prediction tasks, the framework reflects real-world clinical decision-making and avoids information leakage.

The results show that predictive performance is largely driven by structured clinical variables available before anesthesia induction, with intraoperative variables and text-derived features providing complementary but limited additional contributions. These findings highlight the importance of early-stage risk stratification and suggest that most predictive information is already available before surgery.

From an interpretability perspective, the proposed framework combines SHAP-based feature attribution with QAChain-generated explanations to provide both quantitative and human-readable insights into model behavior. Rather than serving as a standalone explanation method, QAChain functions as a translation layer that converts structured feature contributions into clinically interpretable narratives. The observed alignment between QAChain explanations and model-derived feature importance indicates meaningful consistency with dominant predictive signals, while allowing for semantic abstraction and contextual reasoning. This consistency supports the intended interpretability contribution of the explanations, although it does not by itself constitute an objective, clinician-based validation of interpretability or usability.

Overall, this work demonstrates a practical, transparent approach to integrating machine learning and large language models into clinical prediction tasks. By bridging structured modeling and natural-language explanation, the proposed framework provides multi-level explanations intended to enhance interpretability and support more transparent clinical decision-making. Because clinician-based evaluation was beyond the scope of this study, this interpretability benefit should be regarded as a promising reference rather than an objectively validated outcome, and warrants confirmation in future clinician-centered studies. Before broader clinical adoption can be considered, the models also require prospective, multi-center external validation; the present results should therefore be regarded as evidence of internal feasibility rather than established clinical applicability.

## Supplementary material

10.2196/84260 Multimedia Appendix 1 Feature timing classification and temporal availability mapping for model development.

10.2196/84260 Multimedia Appendix 2 Per-variable zero-rate and true missingness summary of the structured feature set.

10.2196/84260 Multimedia Appendix 3 Configuration, prompt template, system instructions, and governance details of the QAChain large language model explanation module.

10.2196/84260 Multimedia Appendix 4 Baseline characteristics of the study population stratified by postoperative vomiting within 24 hours.

10.2196/84260 Multimedia Appendix 5 Supporting analysis of text-derived features, including concept hit rates, postoperative vomiting rate comparisons, signal strength, the coverage funnel, and concept-level statistics.

## Abbreviations

ASA: American Society of Anesthesiologists
CIPONV: clinically significant postoperative nausea and vomiting
DL: deep learning
GA: general anesthesia
KNN: k-nearest neighbor
LightGBM: Light Gradient Boosting Machine
LLM: large language model
ML: machine learning
NaN: not a number
PONV: postoperative nausea and vomiting:
POV 24h: predicting postoperative vomiting within 24 hours
QA: Question Answering
SHAP: Shapley Additive Explanations
TCM: traditional Chinese medicine
TIVA: total intravenous anesthesia
WBC: white blood cells
XAI: explainable AI
XGBoost: Extreme Gradient Boosting
