# Supplementary material for: Prediction of Postoperative Vomiting Within 24 Hours Using Machine Learning With Large Language Model–Enhanced Interpretability: Development and Validation Study
Source: JMIR Med Inform. 2026 Jul 31;14:e84260. doi: 10.2196/84260 (PMC13427058; doi:10.2196/84260)
Supplement: Multimedia Appendix 1 [file medinform-v14-e84260-s001.docx]

**Supplementary Table S-TimingMap.**

Feature timing classification and temporal availability mapping for model development.

| **Variable** | **Timing Category** | **Feature Type** | **Used in Preoperative Model** | **Used in Perioperative Model** | **Exclusion Reason** |
| --- | --- | --- | --- | --- | --- |
| ScheduleNo | Identifier | Structured | No | No | Identifier/timing — not a predictive feature |
| SurgeryDate | Identifier | Structured | No | No | Identifier/timing variable — used for cohort construction or temporal splitting only; not a predictive feature |
| Registration | Identifier | Structured | No | No | Identifier/timing — not a predictive feature |
| ClinicalMicroscopy | Preoperative | Structured | Yes | Yes | – |
| Back | Preoperative | Structured | Yes | Yes | – |
| ASA | Preoperative | Structured | Yes | Yes | – |
| ASA_E | Preoperative | Structured | Yes | Yes | – |
| AnesthesiaStartTime | Identifier | Structured | No | No | Identifier/timing variable — used for cohort construction or temporal splitting only; not a predictive feature |
| AnesthesiaEndTime | Identifier | Structured | No | No | Identifier/timing variable — used for cohort construction or temporal splitting only; not a predictive feature |
| SurgeryStartTime | Identifier | Structured | No | No | Identifier/timing variable — used for cohort construction or temporal splitting only; not a predictive feature |
| SurgeryEndTime | Identifier | Structured | No | No | Identifier/timing variable — used for cohort construction or temporal splitting only; not a predictive feature |
| EnterRoomTime | Identifier | Structured | No | No | Identifier/timing variable — used for cohort construction or temporal splitting only; not a predictive feature |
| LeaveRoomTime | Identifier | Structured | No | No | Identifier/timing variable — used for cohort construction or temporal splitting only; not a predictive feature |
| InductionDosingTime | Identifier | Structured | No | No | Identifier/timing variable — used for cohort construction or temporal splitting only; not a predictive feature |
| PreInductionTime | Identifier | Structured | No | No | Identifier/timing variable — used for cohort construction or temporal splitting only; not a predictive feature |
| PreInductionBP1 | Preoperative | Structured | Yes | Yes | – |
| PreInductionBP2 | Preoperative | Structured | Yes | Yes | – |
| PreInductionPR | Preoperative | Structured | Yes | Yes | – |
| PreInductionSpO2 | Preoperative | Structured | Yes | Yes | – |
| PreInductionTemp | Preoperative | Structured | Yes | Yes | – |
| Age | Preoperative | Structured | Yes | Yes | – |
| BW | Preoperative | Structured | Yes | Yes | – |
| BH | Preoperative | Structured | Yes | Yes | – |
| BMI | Preoperative | Structured | Yes | Yes | – |
| GA | Preoperative | Structured | Yes | Yes | – |
| LMA | Preoperative | Structured | Yes | Yes | – |
| TIVA | Preoperative | Structured | Yes | Yes | – |
| EPI | Preoperative | Structured | Yes | Yes | – |
| NerveBlock | Preoperative | Structured | Yes | Yes | – |
| MaskGA | Preoperative | Structured | Yes | Yes | – |
| DifficultIntubation | Intraoperative | Structured | No | Yes | Intraoperative variable — excluded from Task A (preop leakage prevention) |
| AwakeIntubation | Intraoperative | Structured | No | Yes | Intraoperative variable — excluded from Task A (preop leakage prevention) |
| Oral | Preoperative | Structured | Yes | Yes | – |
| Cuff | Preoperative | Structured | Yes | Yes | – |
| Tracheostomy | Preoperative | Structured | Yes | Yes | – |
| Nasal | Preoperative | Structured | Yes | Yes | – |
| Endo | Preoperative | Structured | Yes | Yes | – |
| BronchCath | Preoperative | Structured | Yes | Yes | – |
| Blocker | Preoperative | Structured | Yes | Yes | – |
| Block | Preoperative | Structured | Yes | Yes | – |
| Temp | Intraoperative | Structured | No | Yes | Intraoperative variable — excluded from Task A (preop leakage prevention) |
| BP1 | Intraoperative | Structured | No | Yes | Intraoperative variable — excluded from Task A (preop leakage prevention) |
| BP2 | Intraoperative | Structured | No | Yes | Intraoperative variable — excluded from Task A (preop leakage prevention) |
| PR | Intraoperative | Structured | No | Yes | Intraoperative variable — excluded from Task A (preop leakage prevention) |
| RR | Intraoperative | Structured | No | Yes | Intraoperative variable — excluded from Task A (preop leakage prevention) |
| ACSugar | Preoperative | Structured | Yes | Yes | – |
| WBC | Preoperative | Structured | Yes | Yes | – |
| RBC | Preoperative | Structured | Yes | Yes | – |
| Hb | Preoperative | Structured | Yes | Yes | – |
| Hct | Preoperative | Structured | Yes | Yes | – |
| PLT | Preoperative | Structured | Yes | Yes | – |
| PT1 | Preoperative | Structured | Yes | Yes | – |
| PT2 | Preoperative | Structured | Yes | Yes | – |
| PTT1 | Preoperative | Structured | Yes | Yes | – |
| PTT2 | Preoperative | Structured | Yes | Yes | – |
| GOT | Preoperative | Structured | Yes | Yes | – |
| GPT | Preoperative | Structured | Yes | Yes | – |
| BUN | Preoperative | Structured | Yes | Yes | – |
| Cr | Preoperative | Structured | Yes | Yes | – |
| Na | Preoperative | Structured | Yes | Yes | – |
| K | Preoperative | Structured | Yes | Yes | – |
| ArtLine | Intraoperative | Structured | No | Yes | Intraoperative variable — excluded from Task A (preop leakage prevention) |
| CVC | Intraoperative | Structured | No | Yes | Intraoperative variable — excluded from Task A (preop leakage prevention) |
| Blanket | Intraoperative | Structured | No | Yes | Intraoperative variable — excluded from Task A (preop leakage prevention) |
| SwanGanz | Intraoperative | Structured | No | Yes | Intraoperative variable — excluded from Task A (preop leakage prevention) |
| EMR | Intraoperative | Structured | No | Yes | Intraoperative variable — excluded from Task A (preop leakage prevention) |
| Complete | Intraoperative | Structured | No | Yes | Intraoperative variable — excluded from Task A (preop leakage prevention) |
| Oral_Fr | Intraoperative | Structured | No | Yes | Intraoperative variable — excluded from Task A (preop leakage prevention) |
| Oral_Fix | Intraoperative | Structured | No | Yes | Intraoperative variable — excluded from Task A (preop leakage prevention) |
| Tracheostomy_Fr | Intraoperative | Structured | No | Yes | Intraoperative variable — excluded from Task A (preop leakage prevention) |
| Nasal_Fr | Intraoperative | Structured | No | Yes | Intraoperative variable — excluded from Task A (preop leakage prevention) |
| BronchCath_Fr | Intraoperative | Structured | No | Yes | Intraoperative variable — excluded from Task A (preop leakage prevention) |
| Department | Preoperative | Structured | Yes | Yes | – |
| Sex | Preoperative | Structured | Yes | Yes | – |
| hr | Intraoperative | Structured | No | Yes | Intraoperative timing variable — excluded from Task A (preop leakage prevention) |
| min | Intraoperative | Structured | No | Yes | Intraoperative timing variable — excluded from Task A (preop leakage prevention) |
| PRECEDEX | Intraoperative | Structured | No | Yes | Intraoperative variable — excluded from Task A (preop leakage prevention) |
| NB | Intraoperative | Structured | No | Yes | Intraoperative variable — excluded from Task A (preop leakage prevention) |
| PCA_y | Preoperative | Structured | Yes | Yes | – |
| Intraoperative_Fentanyl | Intraoperative | Structured | No | Yes | Intraoperative timing variable — excluded from Task A (preop leakage prevention) |
| Intraoperative_Morphine | Intraoperative | Structured | No | Yes | Intraoperative timing variable — excluded from Task A (preop leakage prevention) |
| Intraoperative_XylocaineSpray | Intraoperative | Structured | No | Yes | Intraoperative timing variable — excluded from Task A (preop leakage prevention) |
| Intraoperative_PRECEDEX | Intraoperative | Structured | No | Yes | Intraoperative timing variable — excluded from Task A (preop leakage prevention) |
| Antiemetic_Dexamethasone | Intraoperative | Structured | No | Yes | Intraoperative variable — excluded from Task A (preop leakage prevention) |
| Antiemetic_Aloxi | Intraoperative | Structured | No | Yes | Intraoperative variable — excluded from Task A (preop leakage prevention) |
| PostopAnalgesic_Fentanyl | Postoperative | Structured | No | No | Postoperative — excluded from all models (leakage prevention) |
| PostopAnalgesic_Morphine | Postoperative | Structured | No | No | Postoperative — excluded from all models (leakage prevention) |
| PostopAnalgesic_Dynastat | Postoperative | Structured | No | No | Postoperative — excluded from all models (leakage prevention) |
| PostopAnalgesic_Ketorolac | Postoperative | Structured | No | No | Postoperative — excluded from all models (leakage prevention) |
| PostopAnalgesic_Nalbuphine | Postoperative | Structured | No | No | Postoperative — excluded from all models (leakage prevention) |
| RecoveryRoom_Fentanyl | Postoperative | Structured | No | No | Postoperative — excluded from all models (leakage prevention) |
| RecoveryRoom_Morphine | Postoperative | Structured | No | No | Postoperative — excluded from all models (leakage prevention) |
| RecoveryRoom_Dynastat | Postoperative | Structured | No | No | Postoperative — excluded from all models (leakage prevention) |
| RecoveryRoom_Ketorolac | Postoperative | Structured | No | No | Postoperative — excluded from all models (leakage prevention) |
| RecoveryRoom_Nalbuphine | Postoperative | Structured | No | No | Postoperative — excluded from all models (leakage prevention) |
| NRS | Postoperative | Structured | No | No | Postoperative — excluded from all models (leakage prevention) |
| NRS_Rest | Postoperative | Structured | No | No | Postoperative — excluded from all models (leakage prevention) |
| NRS_Movement | Postoperative | Structured | No | No | Postoperative — excluded from all models (leakage prevention) |
| PCA_UseForHighNRS | Postoperative | Structured | No | No | Postoperative — excluded from all models (leakage prevention) |
| NRS_AfterIntervention | Postoperative | Structured | No | No | Postoperative — excluded from all models (leakage prevention) |
| WoundPain_Rest | Postoperative | Structured | No | No | Postoperative — excluded from all models (leakage prevention) |
| WoundPain_Movement | Postoperative | Structured | No | No | Postoperative — excluded from all models (leakage prevention) |
| SoreThroat | Postoperative | Structured | No | No | Postoperative — excluded from all models (leakage prevention) |
| SoreThroat_Severity | Postoperative | Structured | No | No | Postoperative — excluded from all models (leakage prevention) |
| Hoarseness | Postoperative | Structured | No | No | Postoperative — excluded from all models (leakage prevention) |
| Hoarseness_Severity | Postoperative | Structured | No | No | Postoperative — excluded from all models (leakage prevention) |
| Nausea | Postoperative | Structured | No | No | Postoperative — excluded from all models (leakage prevention) |
| Nausea_0to6h | Postoperative | Structured | No | No | Postoperative — excluded from all models (leakage prevention) |
| Nausea_6hPlus | Postoperative | Structured | No | No | Postoperative — excluded from all models (leakage prevention) |
| Nausea_Day2 | Postoperative | Structured | No | No | Postoperative — excluded from all models (leakage prevention) |
| PCA_UseForNausea | Postoperative | Structured | No | No | Postoperative — excluded from all models (leakage prevention) |
| PCA_UseReason_Nausea | Postoperative | Structured | No | No | Postoperative — excluded from all models (leakage prevention) |
| Vomiting | Postoperative | Structured | No | No | Postoperative — excluded from all models (leakage prevention) |
| Vomiting_0to6h | Postoperative | Structured | No | No | Postoperative — excluded from all models (leakage prevention) |
| Vomiting_6hPlus | Postoperative | Structured | No | No | Postoperative — excluded from all models (leakage prevention) |
| Vomiting_Day2 | Postoperative | Structured | No | No | Postoperative — excluded from all models (leakage prevention) |
| PCA_UseForVomiting | Postoperative | Structured | No | No | Postoperative — excluded from all models (leakage prevention) |
| PCA_UseReason_Vomiting | Postoperative | Structured | No | No | Postoperative — excluded from all models (leakage prevention) |
| Dizziness_Day1 | Postoperative | Structured | No | No | Postoperative — excluded from all models (leakage prevention) |
| Dizziness_Day2 | Postoperative | Structured | No | No | Postoperative — excluded from all models (leakage prevention) |
| Limb_MusclePain | Postoperative | Structured | No | No | Postoperative — excluded from all models (leakage prevention) |
| Headache | Postoperative | Structured | No | No | Postoperative — excluded from all models (leakage prevention) |
| LowerBackPain | Postoperative | Structured | No | No | Postoperative — excluded from all models (leakage prevention) |
| SkinRash | Postoperative | Structured | No | No | Postoperative — excluded from all models (leakage prevention) |
| Pruritus | Postoperative | Structured | No | No | Postoperative — excluded from all models (leakage prevention) |
| Anesthesia_Duration | Intraoperative | Structured | No | Yes | Intraoperative timing variable — excluded from Task A (preop leakage prevention) |
| PlannedProcedure_MeanEmbedding | Preoperative | Text score | Yes | Yes | – |
| MedicalHistoryaverage | Preoperative | Text score | Yes | Yes | – |
| PreopDiagnosis_MeanEmbedding | Preoperative | Text score | Yes | Yes | – |
| PONVMX | Preoperative | Structured | No | No | Postoperative — excluded from all models (leakage prevention) |
| PreopDiagnosis | Preoperative | Text raw | No | No | Raw text — excluded; text-derived score features used instead |
| PlannedProcedure | Preoperative | Text raw | No | No | Raw text — excluded; text-derived score features used instead |
| MedicalHistory | Preoperative | Text raw | No | No | Raw text — excluded; text-derived score features used instead |
| surgery_id | Identifier | Structured | No | No | Identifier/timing — not a predictive feature |
| surgery_date | Identifier | Structured | No | No | Identifier/timing — not a predictive feature |
| anesthesia_start_time | Identifier | Structured | No | No | Identifier/timing — not a predictive feature |
| anesthesia_end_time | Identifier | Structured | No | No | Identifier/timing — not a predictive feature |
| is_duplicate_surgery_record | Identifier | Structured | No | No | Identifier/timing — not a predictive feature |
| master_build_note | Identifier | Structured | No | No | Identifier/timing — not a predictive feature |
| vomit_any_24h | Outcome/Label | Structured | No | No | Postoperative — excluded from all models (leakage prevention) |
| num_interviews_24h | Outcome/Label | Structured | No | No | Postoperative — excluded from all models (leakage prevention) |
| has_followup_24h | Outcome/Label | Structured | No | No | Postoperative — excluded from all models (leakage prevention) |
| followup_missing_flag | Outcome/Label | Structured | No | No | Postoperative — excluded from all models (leakage prevention) |
| first_vomit_time_from_end_surgery | Outcome/Label | Structured | No | No | Postoperative — excluded from all models (leakage prevention) |
| label_pov_24h | Outcome/Label | Structured | No | No | Postoperative — excluded from all models (leakage prevention) |
