# Supplementary material for: Prediction of Postoperative Vomiting Within 24 Hours Using Machine Learning With Large Language Model–Enhanced Interpretability: Development and Validation Study
Source: JMIR Med Inform. 2026 Jul 31;14:e84260. doi: 10.2196/84260 (PMC13427058; doi:10.2196/84260)
Supplement: Multimedia Appendix 2 [file medinform-v14-e84260-s002.docx]

**Feature Missingness and Zero-Value Distribution Report**

*N = 33,460 surgical procedures. All 78 model features (perioperative model) have NaN Rate = 0% after preprocessing.*

**Table 1. Data completeness and zero-value summary by feature type and timing.**

| **Feature Type** | **N** | **NaN Rate** | **Median Zero Rate** | **Max Zero Rate** | **Timing** |
| --- | --- | --- | --- | --- | --- |
| Numeric (continuous) | 25 | 0% | 0.21% | 1.69% (Cr) | Preoperative |
| Binary | 15 | 0% | —a | —a | Preoperative |
| Categorical | 6 | 0% | 0% | 0% | Preoperative |
| **Subtotal: Preoperative** | **46** | **0%** | **—** | **—** |  |
| Numeric (continuous) | 16 | 0% | 25.53% | 99.74% (Morphine) | Intraoperative |
| Binary | 10 | 0% | —a | —a | Intraoperative |
| Categorical | 3 | 0% | 0% | 0% | Intraoperative |
| **Subtotal: Intraoperative** | **29** | **0%** | **—** | **—** |  |
| Text-derived score | 3 | 0% | 0% | 0% | Preoperative |
| **Total** | **78** | **0%** | **—** | **—** |  |

*a Zero rate for Binary variables reflects clinical prevalence of the event (e.g., TIVA used in 13.4% of cases), not data missingness. Binary variables are complete by definition (0 = event absent).*

*N = 33,460. NaN Rate = 0% for all 78 variables. Zero rates for numeric variables indicate cases where the procedure was not performed and the measurement was therefore not recorded.*

**Table 2. Zero-value rate distribution for numeric features (N = 41).**

| **Zero-Value Rate** | **Preop Numeric** | **Intraop Numeric** | **Total** | **Clinical Explanation** |
| --- | --- | --- | --- | --- |
| 0% (complete) | 2 | 0 | 2 | Age, BW — always recorded |
| < 5% | 23 | 6 | 29 | Lab values, vital signs |
| 5–20% | 0 | 2 | 2 | hr, min (anesthesia timing) |
| 20–50% | 0 | 1 | 1 | Intraoperative_Fentanyl |
| ≥ 50% | 0 | 7 | 7 | Airway size variables, Morphine, Xylocaine — only recorded when procedure performed |
| **Total Numeric** | **25** | **16** | **41** | **—** |

*The 7 numeric variables with ≥50% zero values are procedure-specific measurements (airway equipment size: Oral_Fr, Oral_Fix, Tracheostomy_Fr, Nasal_Fr, BronchCath_Fr; intraoperative drug dosage: Intraoperative_Morphine, Intraoperative_XylocaineSpray). Zero values in these variables reflect clinical non-application rather than missing data.*

**Table 3. Imputation strategy by feature type.**

| **Feature Type** | **N** | **Imputation Strategy** | **Note** |
| --- | --- | --- | --- |
| Numeric (continuous) | 41 | Median imputation (training data only) | Applied within each CV fold |
| Binary | 25 | Mode imputation (training data only) | 3 variables have 100% zero values |
| Categorical | 9 | Mode imputation (training data only) | All complete (0% NaN, 0% zero) |
| Text-derived score | 3 | No imputation (rule-based derivation) | All complete |
| **Total** | **78** | **—** | **—** |
