# Supplementary material for: Prediction of Postoperative Vomiting Within 24 Hours Using Machine Learning With Large Language Model–Enhanced Interpretability: Development and Validation Study
Source: JMIR Med Inform. 2026 Jul 31;14:e84260. doi: 10.2196/84260 (PMC13427058; doi:10.2196/84260)
Supplement: Multimedia Appendix 3 [file medinform-v14-e84260-s003.docx]

### B.1 Overview

The QAChain module was designed as a post-hoc explanation component to generate structured, human-readable interpretations of model predictions. To ensure reproducibility and controlled behavior, the LLM component was implemented within a deterministic, constraint-driven framework.

The QAChain module uses a configurable large language model specified via the --llm-model parameter. The default model is resolved from the environment variable LLM_MODEL, with a fallback default of:

**Table B. 1 Configuration of the QAChain LLM module**

| Model | gpt-4o-mini |
| --- | --- |
| Temperature | 0 (deterministic setting) |
| Maximum tokens | 200 |
| API timeout | 60 seconds |

These settings ensure stable and reproducible outputs across repeated runs with identical inputs.

Prompts are constructed programmatically for each individual case based on structured model outputs and SHAP-derived feature attribution. Each prompt includes the predicted risk label and probability, as well as risk-increasing and risk-reducing factors identified from SHAP analysis.

To ensure controlled and clinically appropriate outputs, several constraints are applied during generation. The model is instructed to produce exactly three sentences, to reference only the provided features, and to avoid introducing additional inferences or treatment recommendations. Speculative expressions (e.g., “may suggest” or “could indicate”) are also discouraged. A system message further constrains the model to act as a clinical explanation assistant, report only explicitly provided information, and use plain, clinically interpretable language.

To further ensure reproducibility and transparency, the exact prompting structure used in QAChain is provided below.

| System prompt:  “You are a clinical explanation assistant for postoperative vomiting within 24 hours (POV 24h) risk prediction.  Follow these strict rules:  - Use only the provided factors.  - Do not introduce new medical information.  - Do not hallucinate causes or treatments.  - Be concise, factual, and clinically appropriate.”  User prompt template:  Assessment: {task}  Predicted PONV risk: {risk_label} ({y_prob_pct})  Risk-increasing factors:  {risk_factors}  Mitigating factors:  {mitigating_factors}  Write exactly 3 clinical sentences that:  1. Summarize the overall risk level.  2. Explain the main contributing risk factors.  3. Mention mitigating or protective factors if any.  Constraints:  - Use only the listed factors.  - Do not repeat the same factor.  - Do not add new assumptions.  - Keep statements clinically precise and concise. |
| --- |

### B.2 Deterministic Processing and Feature Selection

To improve consistency and reproducibility, the QAChain pipeline incorporates deterministic preprocessing steps. Risk labels are assigned using predefined thresholds (low: <0.3, moderate: 0.3–0.6, high: ≥0.6), and SHAP features are ranked by absolute contribution. Only the top-ranked features are included in the explanation generation process, ensuring a consistent and controlled input representation for the LLM.

### B.3 Fallback Mechanism

To enhance robustness, a fallback mechanism is implemented. If the LLM service is unavailable (e.g., due to missing API credentials or an API failure), the system generates explanations using predefined deterministic templates. This ensures that the interpretability pipeline remains functional without reliance on external LLM services.

### B.4 Output Validation

Generated explanations are evaluated using rule-based validation checks, including verification of sentence count, detection of restricted content (e.g., treatment-related language), and consistency with the provided feature inputs. Outputs that violate predefined constraints are flagged for inspection.

### B.5 Reproducibility and Governance Considerations

The QAChain module is designed to improve reproducibility through deterministic configuration, structured inputs, and constrained prompting. However, using external LLM services introduces potential limitations, including the need for model updates, API changes, and long-term model drift. These factors may affect output stability over time and should be considered in future deployment and evaluation.
