# Supplementary material for: Prediction of Postoperative Vomiting Within 24 Hours Using Machine Learning With Large Language Model–Enhanced Interpretability: Development and Validation Study
Source: JMIR Med Inform. 2026 Jul 31;14:e84260. doi: 10.2196/84260 (PMC13427058; doi:10.2196/84260)
Supplement: Multimedia Appendix 4 [file medinform-v14-e84260-s004.docx]

**Table A. 1 Baseline Characteristics of the Study Population Stratified by POV 24h Status**

|  | All Cases (N = 33,460) | No POV (n = 29,853) | POV (n = 3,607) |
| --- | --- | --- | --- |
| Patient Physiological Factors | | | |
| Gender (Female) ^a^ | 15118/33460 (45.2%) | 12807/29853 (42.9%) | 2311/3607 (64.1%) |
| Medical History | Unstructured data | Unstructured data | Unstructured data |
| Surgery | Unstructured data | Unstructured data | Unstructured data |
| Preoperative Diagnosis | Unstructured data | Unstructured data | Unstructured data |
| ASA I ^a^ | 866/33460 (2.6%) | 726/29853 (2.4%) | 140/3607 (3.9%) |
| ASA II ^a^ | 15647/33460 (46.8%) | 13602/29853 (45.6%) | 2045/3607 (56.7%) |
| ASA III ^a^ | 16224/33460 (48.5%) | 14832/29853 (49.7%) | 1392/3607 (38.6%) |
| ASA IV ^a^ | 658/33460 (2.0%) | 631/29853 (2.1%) | 27/3607 (0.7%) |
| PreInductionBP1 ^a^ | 144.2 (113–175.4) | 144.0 (112.7–175.3) | 145.1 (114.7–175.5) |
| PreInductionBP2 | 78.7 (62.5–94.9) | 78.7 (62.3–95.1) | 78.7 (63.6–93.8) |
| PreInductionPR ^a^ | 76.3 (58.7–93.9) | 76.5 (58.5–94.5) | 75.5 (60.1–90.9) |
| PreInductionTemp ^a^ | 35.2 (29.3–41.1) | 35.1 (29–41.2) | 35.4 (30.4–40.4) |
| Age ^a^ | 56.5 (39.6–73.4) | 57.5 (40.7–74.3) | 51.9 (35.3–68.5) |
| BW ^a^ | 65.7 (51–80.4) | 66.1 (51.3–80.9) | 63.5 (49.5–77.5) |
| BH ^a^ | 160.2 (139.2–181.2) | 160.4 (138.3–182.5) | 159.4 (144.9–173.9) |
| BMI ^a^ | 24.4 (18.9–29.9) | 24.4 (18.8–30.0) | 24.5 (19.6–29.4) |
| Temp ^a^ | 36.1 (32.2–40) | 36.1 (32.2–40) | 36.1 (32.6–39.6) |
| BP1 ^a^ | 132.1 (107–157.2) | 132.5 (107.3–157.7) | 130.4 (106.1–154.7) |
| BP2 | 78.2 (62.7–93.7) | 78.2 (62.5–93.9) | 78.2 (63.6–92.8) |
| PR ^a^ | 74.6 (57.6–91.6) | 74.9 (57.6–92.2) | 72.6 (57.5–87.7) |
| RR ^a^ | 17.5 (14.5–20.5) | 17.5 (14.4–20.6) | 17.4 (14.8–20) |
| ACSugar | 68 (4.4–131.6) | 67.9 (3.4–132.4) | 68.2 (9.4–127) |
| WBC ^a^ | 7.9 (3.9–11.9) | 8.0 (3.8–12.2) | 7.2 (4.4–10) |
| RBC ^a^ | 4.4 (3.6–5.2) | 4.4 (3.6–5.2) | 4.5 (3.8–5.2) |
| Hb ^a^ | 12.8 (10.5–15.1) | 12.7 (10.4–15.0) | 12.9 (11–14.8) |
| Hct ^a^ | 38.3 (31.9–44.7) | 38.2 (31.6–44.8) | 39.1 (33.9–44.3) |
| PLT ^a^ | 253.6 (162.3–344.9) | 252.1 (158.7–345.5) | 261 (181.6–340.4) |
| GOT ^a^ | 29.3 (0–64.7) | 29.9 (0–68.1) | 25.9 (11.9–39.9) |
| GPT ^a^ | 26.7 (7.5–45.9) | 27 (7.5–46.5) | 25.2 (7.8–42.6) |
| BUN ^a^ | 17.4 (2.2–32.6) | 17.8 (2.2–33.4) | 15.4 (2.7–28.1) |
| Cr ^a^ | 1.3 (0–3.3) | 1.3 (0–3.3) | 1.1 (0–2.8) |
| Na ^a^ | 136.5 (125.9–147.1) | 136.3 (125.2–147.4) | 137.3 (129.9–144.7) |
| K ^a^ | 3.9 (3.3–4.5) | 3.9 (3.3–4.5) | 3.9 (3.4–4.4) |
| Anesthesia-Related Factors | | | |
| TIVA ^a^ | 14200/33460 (42.4%) | 12960/29853 (43.4%) | 1240/3607 (34.4%) |
| EPI ^a^ | 1000/33460 (3.0%) | 926/29853 (3.1%) | 74/3607 (2.1%) |
| Nerve Block ^a^ | 349/33460 (1.0%) | 301/29853 (1.0%) | 48/3607 (1.3%) |
| Mask GA | 4/33460 (0.0%) | 4/29853 (0.0%) | 0/3607 (0.0%) |
| Medication-Related Factors | | | |
| Intraoperative Fentanyl ^a^ | 60.4 (25.8–95.1) | 59.4 (24.1–94.6) | 65.3 (33.9–96.7) |
| Intraoperative Morphine | 3.8 (1.3–6.4) | 4.0 (1.3–6.7) | 3.1 (1.5–4.7) |
| Intraoperative Xylocaine Spray ^a^ | 40.3 (29.2–51.4) | 41.0 (27.9–54.1) | 38.8 (33.9–43.8) |
| Intraoperative PRECEDEX ^a^ | 306/33460 (0.9%) | 281/29853 (0.9%) | 25/3607 (0.7%) |
| Postoperative Analgesics (Fentanyl) ^a^ | 49.8 (32.9–66.8) | 49.5 (32.1–66.9) | 51.4 (36.9–65.8) |
| Postoperative Analgesics (Morphine) ^a^ | 4.7 (3.2–6.2) | 4.7 (3.2–6.2) | 4.7 (3.3–6.2) |
| Postoperative Analgesics (Dynastat) ^a^ | 39.8 (38.0–41.7) | 39.8 (37.9–41.8) | 39.9 (38.3–41.5) |
| Postoperative Analgesics (Ketorolac) ^a^ | 29.9 (27.7–32.0) | 29.8 (27.4–32.3) | 30.0 (28.7–31.3) |
| Postoperative Analgesics (Nalbuphine) ^a^ | 5.9 (3.2–8.6) | 6.1 (3.2–8.9) | 5.6 (3.2–8.0) |
| Surgery-Related Factors | | | |
| Department ^a^ | 25 surgical specialties |  |  |
| Anesthesia Duration ^a^ | 148.0 (19.1–276.9) | 159.3 (57.7–260.9) | 145.7 (12.0–279.4) |
| GA ^a^ | 14768/33460 (44.1%) | 12824/29853 (43.0%) | 1944/3607 (53.9%) |
| LMA ^a^ | 13083/33460 (39.1%) | 11583/29853 (38.8%) | 1500/3607 (41.6%) |
| Difficult Intubation ^a^ | 246/33460 (0.7%) | 226/29853 (0.8%) | 20/3607 (0.6%) |
| Awake Intubation ^a^ | 136/33460 (0.4%) | 130/29853 (0.4%) | 6/3607 (0.2%) |
| Tracheostomy ^a^ | 528/33460 (1.6%) | 510/29853 (1.7%) | 18/3607 (0.5%) |
| Endo ^a^ | 211/33460 (0.6%) | 210/29853 (0.7%) | 1/3607 (0.0%) |
| Bronch Cath ^a^ | 848/33460 (2.5%) | 708/29853 (2.4%) | 140/3607 (3.9%) |
| Blocker ^a^ | 24/33460 (0.1%) | 22/29853 (0.1%) | 2/3607 (0.1%) |
| ArtLine ^a^ | 5667/33460 (16.9%) | 5030/29853 (16.8%) | 637/3607 (17.7%) |
| CVC ^a^ | 1919/33460 (5.7%) | 1789/29853 (6.0%) | 130/3607 (3.6%) |
| Blanket ^a^ | 21979/33460 (65.7%) | 19417/29853 (65.0%) | 2562/3607 (71.0%) |
| Oral Fr ^a^ | 8.5 (6.4–10.6) | 7.9 (5.8–10.0) | 8.6 (6.5–10.7) |
| Tracheostomy Fr ^a^ | 8.4 (4.6–12.2) | 8.7 (4.7–12.7) | 8.4 (4.6–12.2) |
| Nasal Fr ^a^ | 3.5 (2.0–4.9) | 3.3 (2.0–4.6) | 3.5 (2.0–5.0) |
| Bronch Cath Fr ^a^ | 8.4 (7.6–8.8) | 8.1 (7.4–8.8) | 8.5 (7.6–9.3) |

^a Indicates variables included in descriptive baseline comparisons. Variables used for model development are defined separately according to temporal availability in Appendix D. Values are presented as n/N (%) or median (IQR).^
