# Supplementary material for: Prediction of Postoperative Vomiting Within 24 Hours Using Machine Learning With Large Language Model–Enhanced Interpretability: Development and Validation Study
Source: JMIR Med Inform. 2026 Jul 31;14:e84260. doi: 10.2196/84260 (PMC13427058; doi:10.2196/84260)
Supplement: Multimedia Appendix 5 [file medinform-v14-e84260-s005.docx]

### ****C.1 S****upporting analysis of text-derived features

**Analysis of Text-Derived Concept Coverage and Association With POV Risk**

1. Concept hit rate across different clinical text fields.





Fig. 11 Concept hit rate across different clinical text fields

1. Comparison of POV rates between records with and without concept matches.


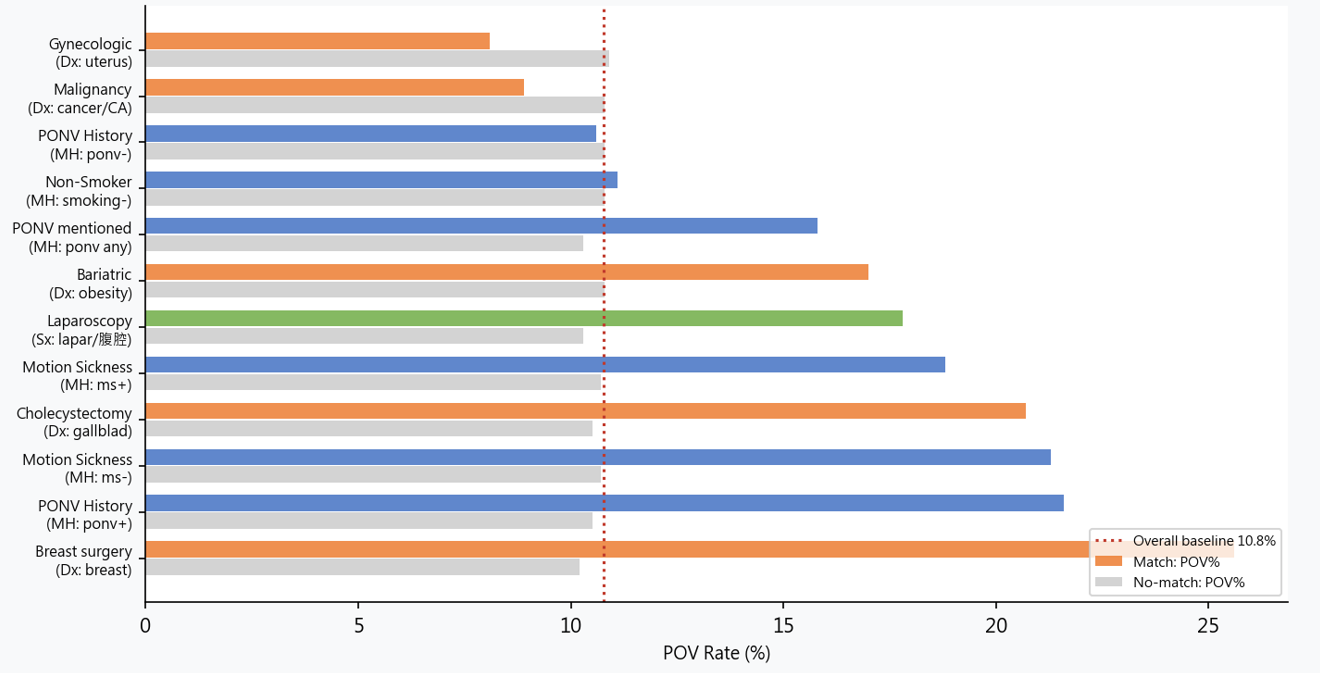


Fig. 12 Comparison of POV rates between records with and without concept matches.

1. Signal strength measured as POV rate difference between matched and non-matched groups.


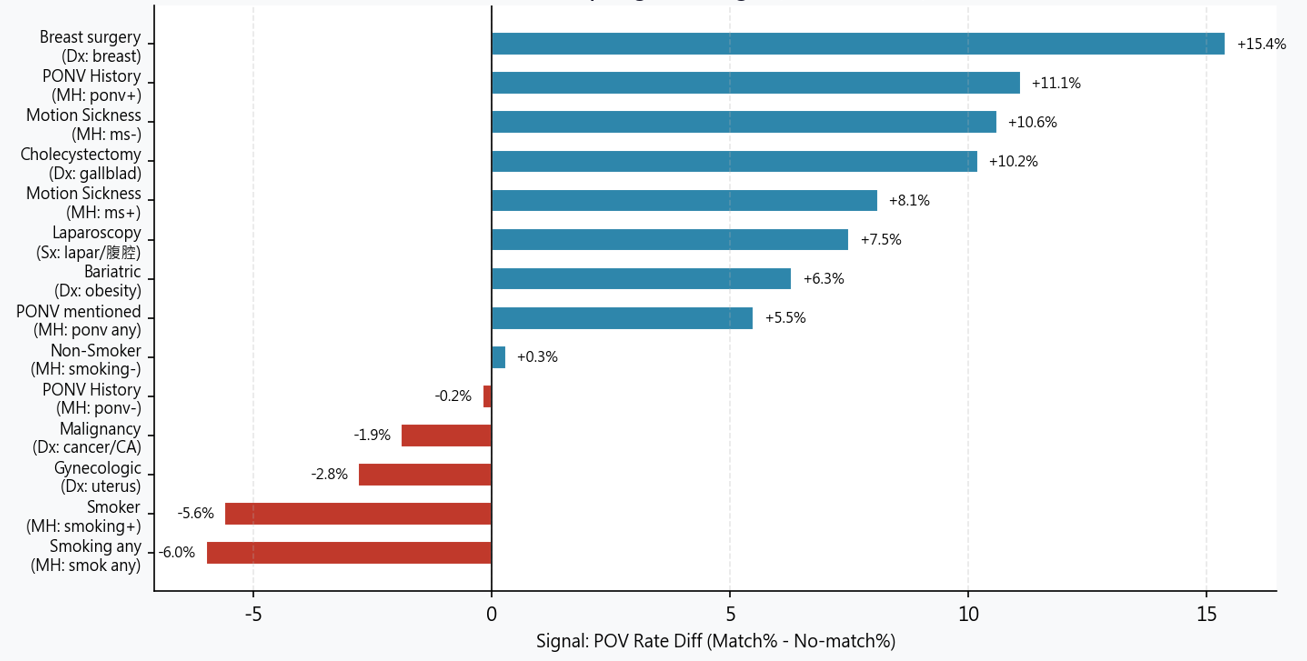


Fig. 13 Signal strength measured as POV rate difference between matched and non-matched groups.

1. Coverage funnel showing the proportion of records with text and matched concepts.


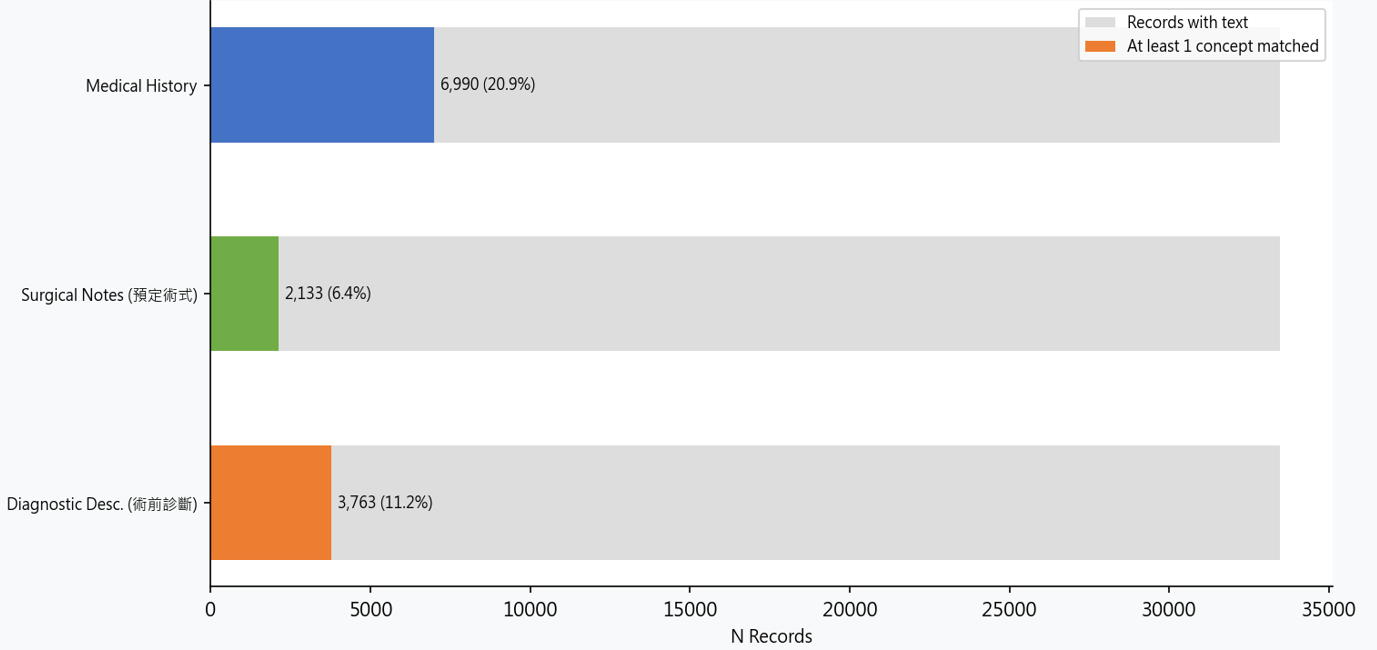


Fig. 14 Coverage funnel showing the proportion of records with text and matched concepts.

1. Summary table of concept-level statistics, including hit rate and associated POV risk.

**Table C. 1 Concept-level statistics of text-derived features and their association with POV risk**

| **Concept** | **Source Field** | **N** | **Hit (%)** | **POV (+)** | **POV (–)** | **Difference (%)** |
| --- | --- | --- | --- | --- | --- | --- |
| Breast surgery | Diagnostic | 1,228 | 3.7 | 25.6 | 10.2 | +15.4 |
| PONV history (+) | Medical history | 753 | 2.3 | 21.6 | 10.5 | +11.1 |
| Motion sickness (+) | Medical history | 108 | 0.3 | 21.3 | 10.7 | +10.6 |
| Cholecystectomy | Diagnostic | 792 | 2.4 | 20.7 | 10.5 | +10.2 |
| Motion sickness | Medical history | 479 | 1.4 | 18.8 | 10.7 | +8.1 |
| Laparoscopy | Surgical notes | 2,133 | 6.4 | 17.8 | 10.3 | +7.5 |
| Bariatric surgery | Diagnostic | 47 | 0.1 | 17.0 | 10.8 | +6.3 |
| PONV mentioned | Medical history | 3,201 | 9.6 | 15.8 | 10.3 | +5.5 |
| Non-smoker | Medical history | 54 | 0.2 | 11.1 | 10.8 | +0.3 |
| PONV history (–) | Medical history | 634 | 1.9 | 10.6 | 10.8 | –0.2 |
| Malignancy | Diagnostic | 639 | 1.9 | 8.9 | 10.8 | –1.9 |
| Gynecologic | Diagnostic | 1,109 | 3.3 | 8.1 | 10.9 | –2.8 |
| Smoker | Medical history | 966 | 2.9 | 5.4 | 10.9 | –5.6 |
| Smoking (any) | Medical history | 3,679 | 11.0 | 5.5 | 11.4 | –6.0 |
